# Genome-driven elucidation of phage-host interplay and impact of phage resistance evolution on bacterial fitness

**DOI:** 10.1038/s41396-021-01096-5

**Published:** 2021-08-31

**Authors:** Pawel Markwitz, Cédric Lood, Tomasz Olszak, Vera van Noort, Rob Lavigne, Zuzanna Drulis-Kawa

**Affiliations:** 1 Department of Pathogen Biology and Immunology, University of Wroclaw, Wroclaw, Poland; 2 Department of Biosystems, Laboratory of Gene Technology, KU Leuven, Leuven, Belgium; 3 Department of Microbial and Molecular Systems, Laboratory of Computational Systems Biology, KU Leuven, Leuven, Belgium; 4 Institute of Biology, Leiden University, Leiden, The Netherlands

**Keywords:** Bacteriophages, Biodiversity

## Abstract

When considering the interactions between bacteriophages and their host, the issue of phage-resistance emergence is a key element in understanding the ecological impact of phages on the bacterial population. It is also an essential parameter for the implementation of phage therapy to combat antibiotic-resistant pathogens. This study investigates the phenotypic and genetic responses of five *Pseudomonas aeruginosa* strains (PAO1, A5803, AA43, CHA, and PAK) to the infection by seven phages with distinct evolutionary backgrounds and recognised receptors (LPS/T4P). Emerging phage-insensitivity was generally accompanied by self and cross-resistance mechanisms. Significant differences were observed between the reference PAO1 responses compared to other clinical representatives. LPS-dependent phage infections in clinical strains selected for mutations in the “global regulatory” and “other” genes, rather than in the LPS-synthesis clusters detected in PAO1 clones. Reduced fitness, as proxied by the growth rate, was correlated with large deletion (20–500 kbp) and phage carrier state. Multi-phage resistance was significantly correlated with a reduced growth rate but only in the PAO1 population. In addition, we observed that the presence of prophages decreased the lytic phage maintenance seemingly protecting the host against carrier state and occasional lytic phage propagation, thus preventing a significant reduction in bacterial growth rate.

## Introduction

*Pseudomonas aeruginosa* is one of the most genetically and phenotypically diverse species driven by frequent horizontal gene transfer (HGT), phase variation, and prophage mobility, thus constitutes a major health threat as an opportunistic human pathogen [1–3]. A thorough understanding of the factors influencing its remarkable adaptability is of critical importance, as the resistance emergence (to chemicals or phages) is an essential parameter to combat the increasing frequency of nosocomial infections caused by *P. aeruginosa* [4]. *P. aeruginosa* isolates carry a large genome ranging between 6 and 7.5 Mbp, and a pangenome of over 54,000 genes. Interestingly, only 665 genes appear to belong to the core genome of the species. Based on core gene phylogeny, five separate *P. aeruginosa* groups may be distinguished [5]. However, the vast majority of sequenced strains originate from groups I (PAO1) and II (PA14) [5–7]. The pangenome of *P. aeruginosa* has been shaped by horizontal gene transfer (HGT), a process that stimulates gene circulation in the bacterial population, and in which transducing phages play a key role by mediating the transduction events. This is evident from the presence of phage genes in about 2% of the core genome and 8% among flexible and unique genes [5]. However, phages also affect the genetics of the bacterial population in a different, more subtle manner, by selecting the phage-resistant mutants during the active propagation in a given population [8]. To counter viral infection, bacteria can inhibit the phage adhesion as the first line of defence, usually by introducing changes/modifications within the receptor recognised by a particular phage [9]. Some resistance mechanisms may be encoded by lysogenic phages and protect the lysogen cell against subsequent infection by closely related phages via superinfection exclusion (Sie) [10]. A large part of the phage-resistance mechanisms/pan-immune system is encoded within chromosomal defence islands, including CRISPR-Cas, R-M, BREX, Abi, DISARM, as well as mobile genetic elements [11–13]. According to this model, individual cells within the population have different combinations of defence mechanisms against different phages [13].

The present study examines the impact of lytic phage infection on the genetic diversity of the *P. aeruginosa* populations and the development of phage resistance. To verify whether a particular phage induces the same type of defence mechanisms in different sensitive bacterial populations and selects for similar genetic modifications, a panel of genetically distinct Pseudomonas phages and a panel of *P. aeruginosa* strains were examined. More specifically, the impact of phage infection at the genetic level and the consequences in terms of the population phenotype as well as the growth rate were assessed and compared. Several questions were asked using our dataset. First, does a particular phage recognising a specific bacterial receptor always select for the cross-resistance to another phage targeting the same macromolecule? Second, do phages recognising the same receptor cause the emergence of the same type of resistant mutants even when they belong to different taxonomy groups? Third, do different strains of *P. aeruginosa* react in a similar fashion to a specific phage infection? Fourth, are the defence response and genome changes correlated with the receptor specificity of infecting phage? Fifth, are there any consequences of gaining phage resistance in terms of population growth efficiency/fitness? Last, does the content of prophages in the genome affect the long-term persistence of lytic phages within the bacterial population?

## Materials and methods

### Bacterial strains and bacteriophages

The *P. aeruginosa* reference panel [14] was screened against the collection of well-characterised phages [15] and seven phages recognising different receptors (LPS or T4P) belonging to different taxonomic clades were chosen to obtain a broad variety of phage selective pressures (Table 1). Finally, five *P. aeruginosa* strains were selected: PAO1, PAK, CHA, and AA43 strains from the phylogeny group I, and strain A5803 from group II, exhibiting high sensitivity to the phage collection. *P. aeruginosa* A5803 was resistant to phage KT28, and AA43 strain showed the resistance to LUZ19 and KTN4 phages (Table 2).

**Table 1:** Characteristics of phages used in this work.

Phage	Taxonomy (Family, Genus)	Genome size	GenBank	Recognised bacterial receptor	Reference
LUZ7*	*Schitoviridae*, *Luzseptimavirus*	74,901 bp	NC_013691	LPS	[53]
KTN6**	*Myoviridae*, *Pbunavirus*	65,994 bp	KP340288	LPS	[54]
KT28**	*Myoviridae*, *Pbunavirus*	66,381 bp	KP340287	LPS	[54]
LUZ19*	*Autographiviridae*, *Phikmvvirus*	43,548 bp	NC_010326	T4P	[55]
phiKZ*	*Myoviridae*, *Phikzvirus*	280,334 bp	AF399011	T4P	[56]
KTN4**	*Myoviridae*, *Phikzvirus*	279,593 bp	KU521356	T4P	[57]
PA5oct**	*Myoviridae*, unclassified	286,783 bp	MK797984	LPS/T4P	[58]

*Laboratory of Gene Technology, KU Leuven, Leuven, Belgium.

**Department of Pathogen Biology and Immunology, Institute of Genetics and Microbiology, University of Wroclaw, Wroclaw, Poland.

**Table 2:** Characteristics of wild-type bacterial strains used in this work.

Strain	Source	Genome size	GenBank accession number	MLST	Population structure [5]	WT resistance to phage collection
PAO1 (ATCC 15692)	Wound	6264,404 bp	NC_002516	549	Group 1	-
PAK*	Non-CF	6395,872 bp	LR657304	693	Group 1	-
CHA*	Cystic fibrosis (CF)	6536,046 bp	CP050149	1919	Group 1	-
A5803*	Community acquired pneumonia	6892,015 bp	CP050147	1567	Group 1	KT28
AA43*	Cystic fibrosis (CF)	6277,301 bp	CP040148	708	Group 2	LUZ19; KTN4

*International *P. aeruginosa* reference panel [14].

### Isolation of phage-resistant clones

For the PAO1 reference strain, bacterial cultures were treated either by individual phage or phage combinations composed of two (KTN6 + LUZ7; KTN6 + phiKZ; KT28 + phiKZ; KTN6 + KTN4) or three viruses (KTN6 + LUZ7 + phiKZ; KTN6 + LUZ7 + KTN4). PA5oct phage was not applied for PAO1 selection but only for phage typing as being already analysed in our previous study [8]. Since other clinical strains differed in phage typing, a reduced number of options were available for experiments: single phages (LUZ7; KTN6; phiKZ; PA5oct) and cocktails (KTN6 + LUZ7; KTN6 + phiKZ; KTN6 + LUZ7 + phiKZ). Bacterial cultures were refreshed in Trypticase Soy Broth (TSB, Oxoid, Basingstoke, UK) to a final concentration of 1 × 10^5^ CFU/ml, transferred to the 96-well peg-lid plate (Nunc, Roskilde, Denmark), and incubated at 37 °C for 24, 48 or 72 h. The peg-lid cover with established biofilms was subsequently transferred to a new plate with TSB medium containing a single phage (or phage combination) at 10^7^ PFU/ml and incubated for 24 h at 37 °C. After treatment, the biofilm was rinsed in PBS buffer and the remaining bacteria were released from the biofilm matrix using an ultrasonic bath (for 15 min). These were immediately plated for colony counting on Trypticase Soy Agar (TSA, Oxoid, Basingstoke, UK). A 10 µl aliquot of the planktonic population was taken simultaneously and plated for the colony count on TSA. The control consisted of the population not treated with phages. From each experimental condition, ten discrete colonies were selected randomly. Each colony was passaged five times on TSA plates to ensure that potential phenotypic changes were stable and further tested for susceptibility to the panel of Pseudomonas lytic phages (Table 1).

### Phage typing with a double-layer plate method

Fresh bacterial cultures were transferred to molten soft agar tubes (TSB, 0.5% technical agar, Oxoid, Basingstoke, UK) and poured into TSA plates. All phages used were diluted in PBS buffer to 10^5^  PFU/ml and spotted (10 µl) on previously prepared bacterial lawns. Plates were incubated for 24 h at 37 °C. Next, the bacterial lawn was visually inspected and the observed plaques indicated the sensitivity of an isolate to a given phage. Phage typing was repeated at least three times. The difference in phage typing patterns allowed us to select isolates for further genome sequencing analysis.

### Growth rate measurement (fitness)

The growth rate of phage-resistant mutants (as well as control strains) was estimated by measuring optical density (OD_600_) kinetics with the starting density of 10^6^  CFU/ml. Cultures prepared in 24-well plates were incubated while shaking for 18 h at 37 °C in a microplate reader (Varioscan Lux, Thermo Scientific). The OD value (λ = 600 nm) was measured at 20-minute intervals. The results of the growth rate measurements were expressed as the cumulated OD values. Each experiment was performed in triplicate. The data was analyzed using the OriginPro 8.5 (OriginLab Corporation, Northampton, Massachusetts, USA). All values were expressed as mean ± SD and significant differences between variations (denoted *p* < 0.001) were assessed with the Tukey test using one-way ANOVA.

### DNA extraction and Illumina sequencing

The genomic DNA was obtained from overnight cultures of the isolates in LB using the PureLink Genomic DNA Mini Kit (Thermo Fisher Scientific). The quality of gDNA samples was assessed using the Qubit dsDNA BR Assay Kit on a Qubit 2.0 device (Thermo Fisher Scientific) and by running the samples on 1% agarose gels with Midori Green (Nippon Genetics Co) staining to assess potential shearing. The gDNA was subsequently prepared for Illumina sequencing using either a TruSeq DNA PCR-Free kit (Illumina) or the NEBNext Ultra DNA Library Prep kit (New England Biolabs) and sequenced on an HiSeq 4000 machine (Illumina) using a paired-end approach (2*151 bp). Wild-type strains gDNA were also sequenced by long-read PacBio RSII technology to fully resolve the chromosome.

### Hybrid genome assembly and annotation of wild type strains

The wild-type strains PAO1, CHA, A5803, and AA43 were assembled de novo by Macrogen, Korea, using the PacBio RS HGAP assembler v3.0 [16], with an additional error correction with Pilon v1.21 [17]. The assemblies were annotated using Prokka v1.14 or NCBI database for PAO1 and PAK [18]. The de novo assembly of CHA was not fully closed, therefore mutations detected within the unknown nucleotide gaps could not be included for further analysis. Prophage annotation was performed with PHASTER [19] and manual curation. All sequences were searched for the presence of CRISPR systems using CRISPRCasFinder [20].

### Population structure

Genome sequences of isolates from different representative groups in the *P. aeruginosa* population were obtained from the NCBI GenBank database (Table S1). They were annotated using Prokka [18] v1.14 and the resulting gff3 files, and those of PAO1, CHA, 15803, AA43, and PAK were used as input to the pangenomics software Roary v3.13 [21]. The software was conFigd to produce a multiple sequence alignment of the core genes (-mafft option) which was subsequently used to reconstruct the phylogeny using FastTree v2 [22].

### Bioinformatics analysis of mutants

The quality of the Illumina sequencing data was assessed using FastQC v0.11.9 [23] and trimmomatic v0.38 [24] for adapter clipping, quality trimming (LEADING:3 TRAILING:3 SLIDINGWINDOW:4:15), and minimum length exclusion (>50 bp). The reads from quality-controlled fastq files were subsequently mapped to the reference genome of PAO1 (NCBI accession NC_002516.2) using the software bwa v0.7.17 [25]. Next, the sequence alignment files were processed using Samtools v1.10 [26] to create sorted BAM files, as well as weeSAM v1.5 to create visual reports of the mapping coverage over the genome of PAO1. The sequencing results were also analyzed using the software pipeline Snippy v4.3.8 [27] to call single nucleotide polymorphisms (SNPs) and insertions/deletions (indels). The analysis pipeline was automated using the tool Snakemake v5.4.4 [28]. The sequences from regions flanking deletions present in the large deletions mutants were analyzed using a custom python script and the software suite MEME v5.2.0 [29].

## Results

**Fig. 1:**
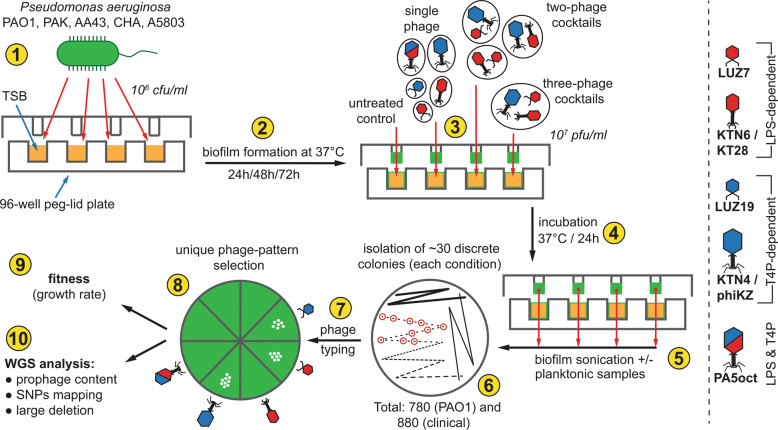
The scheme of experimental pipeline used in this study to examine the impact of lytic phage infection on the *P. aeruginosa* population and the development of phage-resistance. Experiments were conducted as follows: culture preparation (1); biofilm formation (2); phage infection with single or cocktail preparations (3); incubation (4); biofilm and planktonic populations sampling (5); culture plating on TSA agar and isolation of discrete colonies (6); phage typing determination (7); to select isolates with unique patterns (8) for further phenotypic (9) and genome sequencing analyses (10).

The following experimental workflow was implemented to address the main questions raised in our study (Fig. 1).

The *P. aeruginosa* PAO1 reference strain and four other clinical representatives were infected with distinct lytic phages in a single or different cocktail combination. Randomly picked colonies from the surviving cultures were then tested in terms of susceptibility to inoculated phages as well as to the others from the Pseudomonas phages panel (Table 1). We were interested in exploring the broadest clonal variability developed in phage infected *Pseudomonas* population. Therefore, the first phase of the study was focused on examining the phenotypic heterogeneity of PAO1 reference mutants (phage typing) within planktonic and biofilm populations. Since the consequences of introducing lytic phages into the bacterial population are difficult to predict, a representative pool of bacterial clones that have survived infection was sampled. A total of 780 *P. aeruginosa* PAO1 clones were typed with phages (planktonic (320), biofilm populations (400) and 60 control clones). No resistance to phages was observed among the control clones taken from untreated biofilm or plankton. Therefore, three biofilm and three planktonic representatives and the wild-type PAO1 were selected for further genetic and fitness analyses (Table S1). Finally, a pool of 95 isolates has been filtered, representing seventeen different phage susceptibility patterns (Tables S1, 2). This selection was based on the maximum variety of phage-type profiles, without accounting for the origin of the isolate (biofilm/plankton), as the infected planktonic bacteria turned out to be less diverse and all phage types were also present in the biofilm population.

Since we did not aim to analyse the differences of planktonic versus sessile cells response to phage infection but rather look for maximum population heterogeneity, we decided to focus the investigation on the biofilm population for the other clinical strains during the second stage of this research. Accordingly, 880 (30 clones from every condition plus 10 control clones for each strain) isolated colonies from A5803, AA43, CHA, and PA biofilm populations were first subjected to phage typing. No phage resistance was observed among clones taken from phage-untreated samples compared to the wild-type strain. Ultimately, 35 phage-treated colonies, three controls, and the wild-type from each strain were selected for further investigation, resulting in a pool of 156 clones in total (39 × 4 strains) representing over twenty different phage susceptibility patterns (Table S1 and S3).

Do phages always select for cross-resistance to other phages recognising the same bacterial receptor?

The application of monovalent phage against reference PAO1 population generally led to the selection of cross-resistance against phages that recognise the same receptor as the applied one (Table S2). This was observed for 12/17 and 23/24 PAO1 clones isolated after LPS- and T4P-dependent phages treatment, respectively. Similar relation (15/20) was only observed for other clinical cultures infected with phiKZ phage (T4P-dependent) (Table S3). The resistance to both groups of phages was less frequent in monovalent infections (14.5% in PAO1 and 32.5% for other clinical strains) compared to polyvalent infections (61.1%; 33/54) and 51.6% (31/60) for PAO1 and clinical strains, respectively. The use of a cocktail of two phages recognising LPS selected for PAO1 clones resistant only to LPS-dependent phages. In contrast, LPS-dependent phages application was mostly accompanied by the emergence of resistance to phages recognising alternative receptors in clinical strains (28/60 cases).

The introduction of a particular phage into the population did not guarantee the isolation of clones resistant to this phage. This event was recorded in the case of single phages, as well as for polyvalent combinations (23 PAO1 mutants). However, the cross-resistance to other phages recognising the same or both receptors did also occur. Interestingly, LUZ7 and KTN6 phages could still infect surviving clinical populations with a frequency of 23/60 and 44/80, respectively. Indicating that the resistance to LPS-dependent phages in clinical strains was more difficult to develop compared to those impaired by giant viruses, with 11/60 and 1/20 still sensitive to phiKZ and PA5oct phages, respectively. Almost all PAO1 (80/95) and clinical (127/140) clones treated with phages developed resistance to phage PA5oct, whereas the resistance to the entire phage panel emerged regardless of the single or cocktails application.

To conclude, the selection of cross-resistance to other phages recognising the same bacterial receptor was mostly valid in the PAO1 model, whereas the other clinical strains primarily developed the cross-resistance to T4P-dependent phages.

Do phages from different taxonomy groups recognising the same receptor cause the emergence of the same type of resistant mutants? Are the defence response and genome changes correlated with the receptor specificity of infecting phage?

**Fig. 2:**
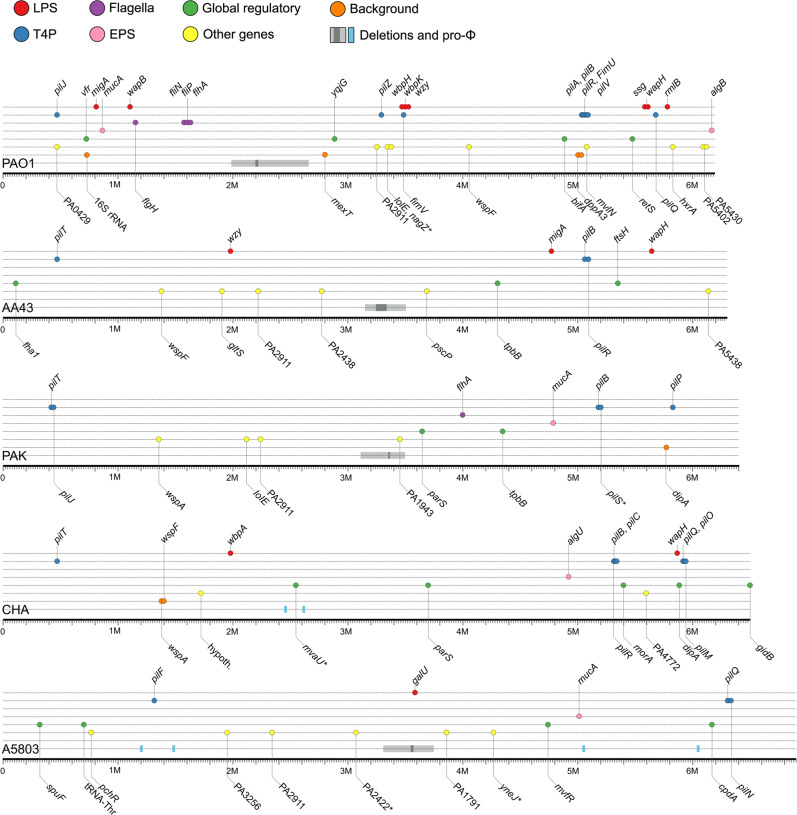
Graphical presentation of genetic changes occurring in the population of *P. aeruginosa* as a result of the infection by selected phages. The colour dots refer to particular gene groups where the point mutations (accumulated results) were recorded within the genomes of examined mutant clones. The lower line contains information on the maximum and minimum size of large deletions (grey bands) and the presence of intact prophages (light blue bands). * means mutation in promoter region of the gene.

**Fig. 3:**
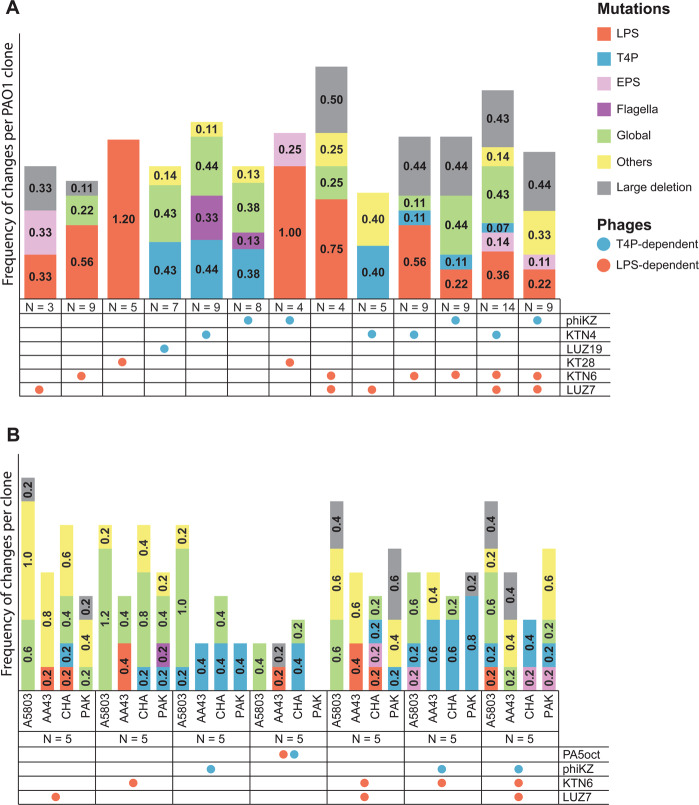
The frequency of genetic changes per clone detected in *P. aeruginosa* strains. Panel (A) represents the PAO1 clones, and panel (B) represents the clinical strains populations. Populations were selected by specific phages targeting LPS (red dots) or T4P (blue dots) as a single treatment or in cocktails. The colour bars refer to particular gene groups where the point mutations were recorded within the genomes of examined mutant clones. N means the number of analysed clones for each strain.

To assess the genetic basis of the resistance selected by phages, we performed single nucleotide polymorphisms (SNPs) and mapping analyses of 102 reference PAO1 clones and 156 clones derived from clinical strains (Figs. 2, 3, Table S2–S4). The wild-type *P. aeruginosa* strains were also re-sequenced with Illumina and PacBio technologies to ascertain their complete genomic background. Missense, nonsense, and frameshift mutation variants were taken into account in the analyses. Mutations that also occurred in control isolates were excluded from further consideration. The remaining mutations were divided into six groups: LPS-related genes, mucoidity-associated genes (EPS production, biofilm formation), T4P-related genes, global regulatory genes, and others (hypothetical or undefined function genes). The comparative analysis showed the presence of point mutations in 64 out of 95 examined PAO1 mutants. The frequency of mutations in PAO1 clones isolated after treatment with single or multiple phages was similar (73% vs 61%, respectively). In most of those isolates, only one gene mutation event was recorded (43%). However, in 23 cases SNPs occurred in two or three genes belonging to different metabolic gene groups. Five PAO1 isolates showed the presence of mutations in two genes from one gene group. The 33 cases of SNPs related to LPS synthesis were found in 29 mutants selected with single LPS-dependent phage preparations or in polyvalent combinations. Among these, the most frequent mutation (21/33 cases) was observed within the *wzy* gene, encoding the B-band O-antigen polymerase [30]. These frequent mutations in the LPS-biosynthesis cluster confirmed the phage resistance results emerging after LUZ7, KT28, and KTN6 phages propagation. In some cases, the LPS gene modification was accompanied by changes in EPS-related genes, leading to a mucoid phenotype. The T4P-dependent phage treatment also led to the selection of specific mutations in genes responsible for T4P expression, but also alterations in flagella-related genes (*flgH, fliN, fliP, flhA*). The mutations in global regulatory genes (most frequent *yqjG* and *vfr*) and “others” gene groups did not show any correlation to the type of phages used.

Apart from point mutations, 23% of phage-resistant PAO1 isolates contained large genomic deletions (23,983 bp–544,729 bp) appeared regardless of the phage-type and cocktail composition used as selective pressure agents. All deletions were located in the same region and despite different starting/ending points, they hold a core element of 19,038 bp. This core element carries the *galU* gene (responsible for LPS biosynthesis), as well as the *hmgA* gene, which causes the accumulation of brown pigment in bacterial cells when absent. Besides, the cumulated deletion range contained a total of 706,374 bp, including many key genes involved in the bacterial metabolism.

Mutations detected in other clinical phage-resistant clones were classified according to the same criteria as in PAO1 (Figs. 2, 3, Table S3, S4). The genome-driven response to phage infection of A5803 was primarily located in global (71%, *cpdA*) and other genes (34%, PA2911); of AA43 in other genes (31%, PA2911); of CHA in T4P (34%) and global genes (34%, *morA*); and of PAK in T4P (25%) and other genes (23%, PA2911). Most of the mutations selected by LPS-dependent phage exposition were found in the global regulatory genes (9–11–25–54%) or “other” genes (17–23–31%), rather than in the LPS biosynthesis locus (0–3–6–17%) depending on the impacted strain (Table S3). That confirmed the phage-typing results where LUZ7 and KTN6 phages remained lytic towards surviving clones. In contrast, the application of phiKZ selected for the cross-resistance to T4P-dependent phages as well as for the genetic modifications in pili-related genes. Mutations in global regulatory and “others” genes show no correlation to the receptor specificity of phages used. Interestingly, a portion of phenotypically phage-resistant clones in each clinical *P. aeruginosa* population (5-9/35 clones) did not reveal any distinguishable genetic modifications. Consistent with PAO1, large genomic deletions were observed in A5803, AA43, and PAK strains ranging between 92,207 bp and 383,693 bp in size, encompassing the *galU* region. The MEME analysis of the regions flanking the deletions did not reveal specific motifs that would indicate recombination events. Interestingly, the unique large deletion found in CHA strain (15,126 bp) turned out to be the induced ssDNA filamentous Pf1-like phage.

Summarising the analyses one might say that phages from different taxonomy groups recognising the same receptor generally cause the emergence of a similar type of resistance within a particular strain. However, the defence response and genome changes correlated with the receptor specificity of infecting phage differ in a strain-dependent manner.

Do different strains of *P. aeruginosa* react similarly to a specific phage infection?

**Fig. 4:**
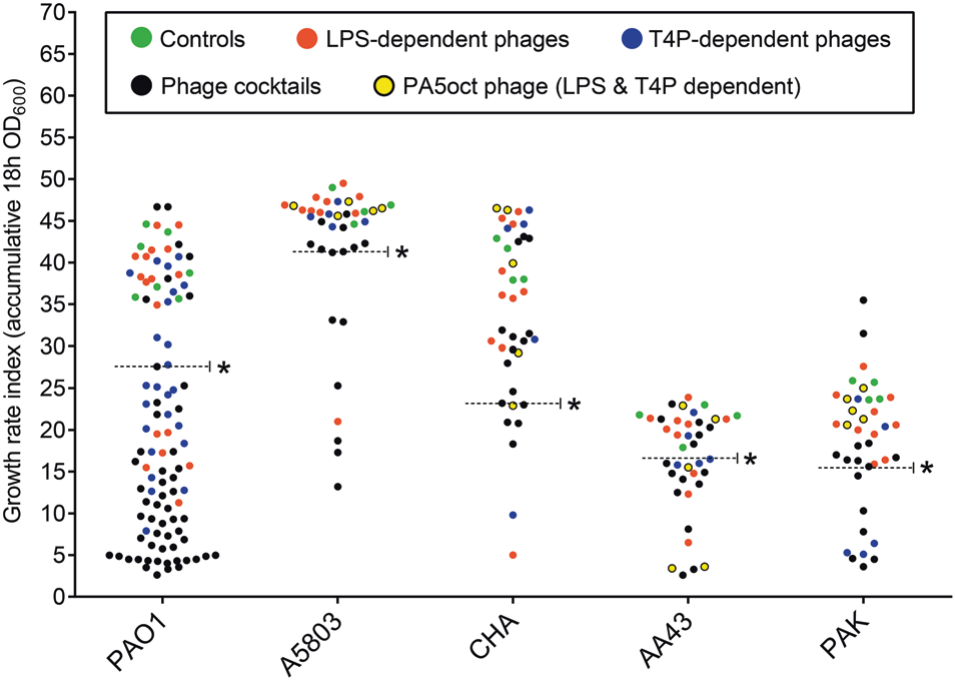
The population growth efficiency as an indicator for bacterial fitness expressed as the cumulative OD_600_ values of 18 h culture at 37 °C measured at 20-minute intervals. Dots represent the growth of particular clones: the wild-type and control clones (green dots); mutants selected by LPS-dependent phage (red dots); mutants selected by T4P-dependent phage (blue dots); mutants selected by LPS/T4P-dependent PA5oct phage (orange dots); mutants selected by phage cocktail (black dots). * statistically different cumulative OD value compared to phage-untreated pool (*p* < 0.001).

The next step aimed to assess the impact of gaining phage resistance in terms of population growth efficiency as an indicator for bacterial fitness. Three of the examined wild-type strains (PAO1, A5803, and CHA) have a naturally rapid growth rate, while the other two (AA43 and PAK) display moderate growth rates. For this reason, the final results are expressed as the cumulated OD_600_ (Fig. 4, Table S2, S3). Overall, the majority of PAO1 mutants (61/95; 64%, *p* < 0.001) showed a significant decrease in growth rate compared to the untreated population. The phage-infected *P. aeruginosa* AA43 behaved similarly to the PAO1 population with 51% of clones with significantly lowered multiplication efficiency. In contrast, the phages selected only 26%, 20%, and 29% of clones exhibiting reduced growth from *P. aeruginosa* A5803, CHA, and PAK populations, respectively. In most cases, these changes were associated with the occurrence of large genomic deletion, the persistence of phage DNA in the genome, or the combination of both. The correlation analysis of the phage-typing profile versus the reduction of growth rate (Table S2, S3) revealed statistically significant differences (*p* > 0.001) for the clones resistant to 6–7 phages but only in the PAO1 group. Moreover, only the selection done by phage cocktails gave a statistically significant reduction of bacterial growth (*p* > 0.001), while no differences were observed regarding groups treated with single LPS- or T4P- dependent phages. In contrast to the PAO1 reference strain, the statistical analyses conducted in the A5803, AA43, CHA, and PAK strains did not show any differences in terms of phage-typing profile nor phage-type selection pressure versus the population fitness reduction (growth rate).

Does prophage content in the genome affect the long-term persistence of lytic phages within the bacterial population?

This final question focused on the content of prophages versus the lytic phage maintenance in the infected population (carrier state). It should be emphasised that the carrier state was considered only when a full contig of phage DNA was present in the population phenotypically resistant to the infection by this particular phage. We were interested to assess differences in lytic phage DNA maintenance in *P. aeruginosa* strains versus the number and variability of temperate phages incorporated in the bacterial genome. The whole-genome analyses revealed that the only sequences of phage origin common to all strains encode pyocins with the similarity to phage phiCTX in A5803 and CHA strains while to phage YMC11/02/R656 in the remaining strains (Table S5). All strains, except AA43, contained a complete or incomplete genome of the filamentous phage Pf1, and only A5803 and CHA genomes contained intact dsDNA prophages. Two A5803 intact prophages showed very high sequence similarity to siphoviruses (A5803#1 → phage JBD25; A5803#4 → phage phi297), whereas the third and fourth were homologous to myoviruses (A5803#3 → *Escherichia* phage vB_EcoM_ep3; A5803#5 → phiCTX phage). The CHA#1 prophage was homologous to JBD18 siphovirus, and the CHA#2 prophage to phiCTX myovirus. The side-by-side comparisons of dsDNA prophage sequences revealed a very low similarity (73% at most for A5803#5 and CHA#2 prophages). In strain AA43, no phage genetic elements were found by PHASTER or manual curation (Table S5).

The genomic analysis of de novo assembled mutants showed that 69/70 isolates of A5803 and CHA strains did not maintain the DNA of lytic phages used for population treatment (no carrier state) (Table 3). On the other hand, this phenomenon was common in clones selected from the intact prophage-free *P. aeruginosa* strains. In the PAK mutants, the carrier state appeared in 8/35 clones, AA43 mutants contained phage DNA in 11/35 cases, and PAO1 mutants in 75/125 clones. Based on the frequency of individual lytic phage DNA detection in mutants, especially in those originating from PAO1, jumbo phages (PA5oct, phiKZ, and KTN4) appeared to be much more efficient in the long-term survival/maintenance (71/75 cases) in the bacterial population, compared to smaller phages.

**Table 3:** Maintenance of phage DNA after treatment in *P. aeruginosa* phage-resistant isolates versus intact prophages in the wild-type strains.

Strain	Maintenance of phage DNA after infection								Intact prophages in WT
	LUZ7	KTN6	KT28	LUZ19	KTN4	phiKZ	PA5oct	Total	
A5803	0/15	0/20	-	-	-	0/15	0/5	0/35	4
CHA	0/15	1/20	-	-	-	0/15	0/5	1/35	2
AA43	2/15	5/20	-	-	-	2/15	2/5	11/35	0
PAK	0/15	1/20	-	-	-	7/15	0/5	8/35	0
PAO1	2/35	2/54	0/9	0/7	27/37	23/29	21/30*	75/125	0

*Data from the previous study [8].

## Discussion

Although some reports have focused on the impact of phage infection in *P. aeruginosa* population, (mostly PA1 and PAO1 strains) [31–35], no study has addressed the complexity of changes taking place within the bacterial population under the phage pressure in a diverse set of clinical isolates. Within a context of the genetic heterogeneity of *P. aeruginosa* population [5, 6], it is important to trace how phages shape the genome of strains belonging to other phylogenetic groups and whether the changes observed for PAO1 are also reflected in other strains. Therefore, this study aimed to verify the phenotypic and genetic response of five *P. aeruginosa* strains (PAO1, A5803, AA43, CHA, and PAK) to the infection of four phages distinct in taxonomic position and genome size (podoviruses, myoviruses, as well as three jumbo phages), all containing characterised receptors (LPS or T4P). The examination of a large number of clones by sequencing (~250), demonstrates the phage impact on many population-modulating factors. However, these effects may coincide or counteract each other, which requires careful analysis and interpretation of such findings. Specifically, our model allowed us to answer several questions.

### Phages recognising a specific bacterial receptor do not always select for the cross-resistance to another phage targeting the same macromolecule

In general, the *P. aeruginosa* clones showed a wide diversity of phage susceptibility patterns in a strain-dependent manner (over 17 phage types). In most cases, T4P-dependent phages caused cross-resistance to other phages recognising the same receptor. Yet surprisingly, LPS-dependent podovirus LUZ7 and siphovirus KTN6, and less often the jumbo phage phiKZ contributed to cross-resistance to phages targeting the same as well as an alternative receptor, but not always to itself, remaining lytic to infected population. It was especially noticeable in the treatment of clinical strains. Such differences can be based on the adhesion mechanism developed by phylogenetically distinct phages and the mechanisms of receptor modification could be heterogenous even within single strains [2]. On the other hand, the jumbo phage PA5oct was the most often targeted by bacterial self and cross-resistance mechanisms, perhaps due to the complexity of the adhesion process (two required receptors LPS + T4P) as well as a previously hypothesised pseudolysogeny [8].

### The bacterial response to phage-infection differs at the genomic level between strains

As the resistance phenotype may have a different genetic background (mutations directly related to the receptor genes, mutations in other genes, phase variation, as well as large genome deletion) or be based on the protein expression level, one might expect variability in this aspect [36–38]. The frequency of genetic changes combined with a varied efficiency of the DNA repair system implies a clonal diversity among *P. aeruginosa* isolates, especially during the colonisation of CF patients [39].

Although *P. aeruginosa* strains tested in this study were genetically distinct, the predation of lytic phages selected clones with similar genome modifications in LPS or type IV fimbriae receptor biosynthesis clusters and large genome deletions. Nevertheless, the occurrence of individual genetic events varied between strains. The most frequent modification in the LPS biosynthesis cluster in PAO1 was located in *wzy* and *ssg* genes whereas in clinical strains were accumulated in the *wzy* and *wapH* genes. The highest number of mutations in T4P genes was observed for all strains within the *pilB*, *pilQ*, *pilR* genes, and especially in *pilT* for the clinical strains. The higher discrepancy between strains was detected in the category of “global regulatory genes”, in which *yqjG* and *vfr* genes were changed in the PAO1 reference population in contrast to mostly *cpdA* and *parS* in clinical representatives. Although it is difficult to accurately predict the impact of these changes at the level of the bacterial cell biology, mutations detected in clinical strains were also observed in *P. aeruginosa* isolates from cystic fibrosis patients [40, 41].

We confirmed previous studies that point mutations impairing LPS production do not affect the sensitivity of bacteria to T4P-dependent phages [31]. On the other hand, the impact of LPS-targeting phages propagating in clinical populations differs compared to the PAO1 population case. Infection by LUZ7 and KTN6 phages was most often accompanied by mutations in the “global regulatory genes” and “other” genes. Moreover, those phages remained lytic against selected phage-resistance clones in 38% and 55%, respectively.

Lytic phage infection, as well as antibiotic treatment, may be accompanied by the loss of a large genome section (over 200 Kbp) [32, 33, 42, 43] and are often found among *P. aeruginosa* strains isolated from cystic fibrosis patients [40]. The deletion of *galU*, *mexXY*, and *hmgA* located in this region results in inhibition of the LPS O-chain production, loss of efflux pumps, and accumulation of homogentisic acid (brown pigment), in addition to increased resistance to pyocins and oxidative stress [44]. The formation of these large deletions is associated with the MutL protein, which by default is involved in DNA mismatch repair processes [34]. Although in our study the large deletions always contained *galU* and *hmgA* genes, we were not able to detect any specific sequences flanking the missing fragments of phage-resistant clones to support a recombination event. Moreover, each deletion contained a different gene set even if the size appeared to be similar. The MutL-dependent mechanism is likely a widespread strategy to avoid lytic phage predation, and our experiments confirmed the same response of the *galU* region deletions in all examined *P. aeruginosa* strains. However, CHA is an exception, as this strain reacted to phage-predation-stress by inducing the prophage in the same way as observed previously [33]. Literature suggests that large deletions are associated with general cellular stress but we found these occurred only after LPS-dependent phages treatment separately or in combinations. This indicates a significant selection pressure induced by LPS-dependent phages on the *P. aeruginosa* population and the large deletion-bearing clones displayed low competitiveness in the medium.

### The phage-imposed reduction in bacterial population fitness (growth rate) is significant only for the PAO1 strain

As phage receptors are bacterial virulence factors, their loss or modification can result in a reduction of bacterial fitness. Therefore, cross-resistance to phages recognising different receptors is only beneficial for a limited period, since it impairs the adaptability of bacteria [35, 45]. Most reports state that genotypic and phenotypic changes occurring in the bacterial population impacted by phage predation usually lead to a reduction in bacterial fitness and, consequently, facilitates the clearing of the infection by the immune system. Moreover, these changes often correspond to an increase in the sensitivity of the bacterial population to certain antibiotics, which makes combined therapy more effective [33, 46, 47]. In the therapeutic context, the phage resistance correlation to bacterial fitness is a crucial issue.

Our study revealed that the most common reasons for the growth rate decrease were the large genome deletion and phage persistence in the bacterial population. Nevertheless, we found that an effective and significant reduction of bacterial growth was observed for multi-phage resistance profile and the application of double or triple phage cocktails, but it was relevant to the PAO1 population only. Although clinical strains tested in this study developed the resistance to phages, the consequences in terms of the decreased growth rate were not significant. Genome-driven elucidation of phage-host interplay and impact on bacterial fitness in clinical *P. aeruginosa* strains might suggest that during the co-evolution pathway bacteria have learned how to control/balance the susceptibility to phage infection and accomplish it not only by the direct modification of genes responsible for specific surface receptor biosynthesis but rather by the global regulation of their cell metabolism. The low frequency of mutations in LPS-related genes, detected in our study, indicates the prevention of specific features crucial for clinically evolved strain. This phenomenon could be further investigated in a wider context of bacterial virulence and by complementing the list of genetic changes with those from larger structural variation analyses (transposon activity, prophage mobility, mobile islands) based on long reads sequencing data [48].

### The content of prophages might influence the lytic phage carriage state in the infected bacterial population

The presence of prophages in the host genome usually protects from the infection by other, closely related phages under superinfection exclusion mechanisms (Sie) [49, 50]. However, we consider that aspect differently as the wild-type hosts we used in our model were sensitive to the panel of infecting lytic phages. We aimed to verify the hypothesis that lytic phages could be maintained in the resistant population in pseudolysogeny events and whether this depends on the composition of prophages inhabiting the bacterial host (Sie mechanisms). Phages can propagate in the bacterial population expressing a variety of modified receptors targeted by the phage which reflects the heterogeneity of *P. aeruginosa* and the portion of persister cells [51]. This can be observed especially in the sessile population living within the biofilm [52]. Another possible explanation is the model of pseudolysogeny of lytic phages described previously [31, 43].

In our experimental model, we assumed the lytic phage carriage state only when the genome assembly techniques resulted in the recovery of a full phage DNA contig in the phenotypically resistant population to this particular phage. Although our experimental design requires a cautious interpretation, the analyses suggest that the maintenance of lytic phage was correlated to the set of prophages present within the host genome. For example, the prophage-free AA43 strain was able to maintain the DNA of all phages used in the study. In contrast, all A5803 clones (35) carrying four intact prophages were free from the lytic phage DNA content. Moreover, we noted the relationship between the composition of prophages and the type of lytic phage maintained in the population. PAO1 and PAK strains, hosting only filamentous phage Pf1 were colonised mainly by phiKZ-like phages, whereas prophage-free AA43 strain was usually colonised with LUZ7 or KTN6 phages. This observation was further supported by the prevalence of pseudolysogeny, which varied from 0-1/35 for CHA and A5803 to the high values of 23%, 31%, and 60% for PAK, AA43, and PAO1, respectively. Therefore, despite the initial sensitivity of the strain to a given phage, the prophages composition could protect against pseudolysogeny, thus presenting the benefits of the parasite-host interactions [49, 50]. The significant correlation between the reduced fitness (growth rate) in the case of the lytic phage carrier state might explain the importance of prophage protection given to their hosts. However, we recognise that the host-parasite interplay might be very complex and might reveal additional host mechanisms that could be responsible for the maintenance (or inhibition) of lytic phage. This could be studied in the future by curing prophages from otherwise isogenic strains and verifying how the population parameters, including the lytic phage carrier state are impacted.

## Supplementary information

Table S1

Table S2

Table S3

Table S4

Table S5
