# Supplementary material for: Genome-driven elucidation of phage-host interplay and impact of phage resistance evolution on bacterial fitness
Source: ISME J. 2021 Aug 31;16(2):533–42. doi: 10.1038/s41396-021-01096-5 (PMC8776877; doi:10.1038/s41396-021-01096-5)
Supplement: Supplementary file 2 — Table S2 [file 41396_2021_1096_MOESM2_ESM.docx]

**Table S2**. Characteristics of *P. aeruginosa* PAO1 control and phage-resistant isolates

| **#** | **STRAIN NAME** | **CUMULATED OD_600_ VALUES**  **OF 18H CULTURE** | **PHAGE SUSCEPTIBILITY** | | | | | | | **PHAGE DNA** | **REGION AFFECTED WITH MUTATION** | | | | | | | | **LARGE DELETION [bp]** |
| --- | --- | --- | --- | --- | --- | --- | --- | --- | --- | --- | --- | --- | --- | --- | --- | --- | --- | --- | --- |
|  |  |  | **LPS phages** | | | **T4P phages** | | |  |  |  |  |  |  |  |  |  |  |  |
|  |  |  | **LUZ7** | **KTN6** | **KT28** | **LUZ19** | **KTN4** | **phiKZ** | **PA5oct** |  | **LPS** | **EPS** | **T4P** | **FLAGELLA** | **GLOBAL** | | **OTHER** | |  |
| **1** | **PAO1_WT** | **43.8** |  |  |  |  |  |  |  |  |  |  |  |  |  | |  | |  |
| **2** | **PAO1-K-24-A** | 38.7 |  |  |  |  |  |  |  |  |  |  |  |  | *mexT* | | *dppA3*; p. p. dppA3; p. p. 16S rRNA | |  |
| **3** | **PAO1-K-48-A** | 37.1 |  |  |  |  |  |  |  |  |  |  |  |  | *mexT* | | *dppA3*; p. p. dppA3; p. p. 16S rRNA | |  |
| **4** | **PAO1-K-72-A** | 35.8  35.66715 |  |  |  |  |  |  |  |  |  |  |  |  | *mexT* | | *dppA3*; p. p. dppA3; p. p. 16S rRNA | |  |
| **5** | **PAO1-K-P-B** | 35.7 |  |  |  |  |  |  |  |  |  |  |  |  | *mexT* | | p. p. dppA3 | |  |
| **6** | **PAO1-K-P-C** | 43.7 |  |  |  |  |  |  |  |  |  |  |  |  | *mexT* | | p. p. dppA3 | |  |
| **7** | **PAO1-K-P-D** | 41.9 |  |  |  |  |  |  |  |  |  |  |  |  | *mexT* | | p. p. dppA3; p. p. 16S rRNA | |  |
| **8** | **PAO1-LUZ7-48-K** | 37.7 | **●** |  |  |  |  |  |  |  |  | *mucA* |  |  | *mexT* | | p. p. dppA3; p. p. 16S rRNA | |  |
| **9** | **PAO1-LUZ7-P-F** | 11.3 | **●** |  |  |  |  |  |  |  |  |  |  |  | *mexT* | | p. p. dppA3; p. p. 16S rRNA | | 291 281 |
| **10** | **PAO1-LUZ7-P-M** | 38.3 | **●** |  |  |  |  |  |  |  | *wbpH* |  |  |  | *mexT* | | p. p. dppA3; p. p. 16S rRNA | |  |
| **11** | **PAO1-KTN6-24-C** | 40.8 |  | **●** |  |  |  |  |  |  | *wzy* |  |  |  | *mexT* | | p. p. dppA3; p. p. 16S rRNA | |  |
| **12** | **PAO1-KTN6-24-D** | 40.7 |  | **●** |  |  |  |  |  |  | *wzy* |  |  |  | *mexT* | | p. p. dppA3; p. p. 16S rRNA | |  |
| **13** | **PAO1-KTN6-48-J** | 17.3 |  | **●** |  |  |  |  |  |  | *rmlB* |  |  |  | *yqjG* | *mexT* | p. p. dppA3. p. p. 16S rRNA | |  |
| **14** | **PAO1-KTN6-72-E** | 15.7 |  | **●** |  |  |  |  |  |  |  |  |  |  | *mexT* | | p. p. dppA3 | |  |
| **15** | **PAO1-KTN6-72-G** | 34.9 |  | **●** |  |  |  |  |  |  |  |  |  |  | *mexT* | | p. p. dppA3; p. p. 16S rRNA | |  |
| **16** | **PAO1-KTN6-72-J** | 44.4 |  | **●** |  |  |  |  |  |  | *wzy* |  |  |  | *mexT* | | p. p. dppA3; p. p. 16S rRNA | |  |
| **17** | **PAO1-KTN6-P-E** | 19.7 |  | **●** |  |  |  |  |  | KTN6 | *wzy* |  |  |  | *mexT* | | p. p. dppA3; p. p. 16S rRNA | |  |
| **18** | **PAO1-KTN6-P-H** | 15.5 |  | **●** |  |  |  |  |  |  |  |  |  |  | *mexT* | | p. p. dppA3; p. p. 16S rRNA | | 275 941 |
| **19** | **PAO1-KTN6-P-J** | 41.5 |  | **●** |  |  |  |  |  |  |  |  |  |  | *yqjG* | *mexT* | p. p. dppA3. p. p. 16S rRNA | |  |
| **20** | **PAO1-KT28-24-B** | 38.0 |  |  | **●** |  |  |  |  |  | *wapB; wzy* |  |  |  | *mexT* | | p. p. dppA3 | |  |
| **21** | **PAO1-KT28-48-A** | 19.5 |  |  | **●** |  |  |  |  |  |  |  |  |  | *mexT* | | *dppA3*; p. p. dppA3 | |  |
| **22** | **PAO1-KT28-48-B** | 38.6 |  |  | **●** |  |  |  |  |  | *wzy* |  |  |  | *mexT* | | *dppA3*; p. p. dppA3 | |  |
| **23** | **PAO1-KT28-72-H** | 44.5 |  |  | **●** |  |  |  |  |  | *migA; wzy* |  |  |  | *mexT* | | *dppA3*; p. p. dppA3; p. p. 16S rRNA | |  |
| **24** | **PAO1-KT28-P-J** | 41.6 |  |  | **●** |  |  |  |  |  | *wzy* |  |  |  | *mexT* | | p. p. dppA3; p. p. 16S rRNA | |  |
| **25** | **PAO1-LUZ19-24-B** | 38.4 |  |  |  | **●** |  |  |  |  |  |  | *pilQ* |  | *yqjG* | *mexT* | p. p. dppA3 | |  |
| **26** | **PAO1-LUZ19-24-E** | 40.7 |  |  |  | **●** |  |  |  |  |  |  |  |  | *mexT* | | p. p. dppA3; p. p. 16S rRNA | |  |
| **27** | **PAO1-LUZ19-48-K** | 40.2 |  |  |  | **●** |  |  |  |  |  |  |  |  | *mexT* | | p. p. dppA3; p. p. 16S rRNA | |  |
| **28** | **PAO1-LUZ19-48-L** | 39.6 |  |  |  | **●** |  |  |  |  |  |  |  |  | *mexT* | |  | |  |
| **29** | **PAO1-LUZ19-P-AA** | 35.3 |  |  |  | **●** |  |  |  |  |  |  | *pilA* |  | *yqjG* | *mexT* | *hxrA* | p. p. dppA3; p. p. 16S rRNA |  |
| **30** | **PAO1-LUZ19-P-AG** | 36.5 |  |  |  | **●** |  |  |  |  |  |  |  |  | *mexT* | | p. p. dppA3; p. p. 16S rRNA | |  |
| **31** | **PAO1-LUZ19-P-Z** | 37.3 |  |  |  | **●** |  |  |  |  |  |  | *pilQ* |  | *retS* | *mexT* | p. p. dppA3; p. p. 16S rRNA | |  |
| **32** | **PAO1-KTN4-24-B** | 14.3 |  |  |  |  | **●** |  |  | KTN4 |  |  |  |  | *vfr* | *mexT* | p. p. dppA3; p. p. 16S rRNA | |  |
| **33** | **PAO1-KTN4-24-F** | 24.8 |  |  |  |  | **●** |  |  | KTN4 |  |  |  | *fliP* | *vfr* | *mexT* | p. p. dppA3 | |  |
| **34** | **PAO1-KTN4-72-B** | 25.3 |  |  |  |  | **●** |  |  | KTN4 |  |  |  |  | *vfr* | *mexT* | p. p. dppA3; p. p. 16S rRNA | |  |
| **35** | **PAO1-KTN4-72-D** | 20.1 |  |  |  |  | **●** |  |  | KTN4 |  |  | *fimV* | *flgH* | *mexT* | | p. p. dppA3; p. p. 16S rRNA | |  |
| **36** | **PAO1-KTN4-72-F** | 12.8 |  |  |  |  | **●** |  |  | KTN4 |  |  |  |  | *vfr* | *mexT* | p. p. dppA3. p. p. 16S rRNA | |  |
| **37** | **PAO1-KTN4-72-I** | 36.8 |  |  |  |  | **●** |  |  |  |  |  | *pilQ* |  | *mexT* | | p. p. dppA3; p. p. 16S rRNA | |  |
| **38** | **PAO1-KTN4-P-G** | 18.4 |  |  |  |  | **●** |  |  | KTN4 |  |  | *pilV* |  | *mexT* | | PA5430 | *dppA3*; p. p. dppA3 |  |
| **39** | **PAO1-KTN4-P-K** | 31.0 |  |  |  |  | **●** |  |  | KTN4 |  |  |  | *flhA* | *mexT* | | *dppA3*; p. p. dppA3; p. p. 16S rRNA | |  |
| **40** | **PAO1-KTN4-P-P** | 27.8 |  |  |  |  | **●** |  |  |  |  |  | *pilB* |  | *mexT* | | p. p. dppA3; p. p. 16S rRNA | |  |
| **41** | **PAO1-phiKZ-24-D** | 7.9 |  |  |  |  |  | **●** |  | PhiKZ |  |  | *pilR* |  | *yqjG* | *mexT* | p. p. dppA3; p. p. 16S rRNA | |  |
| **42** | **PAO1-phiKZ-24-H** | 38.8 |  |  |  |  |  | **●** |  | PhiKZ |  |  |  |  | *mexT* | | p. p. dppA3; p. p. 16S rRNA | |  |
| **43** | **PAO1-phiKZ-24-J-5** | 24.2 |  |  |  |  |  | **●** |  | PhiKZ |  |  | *pilJ* |  | *mexT* | | *dppA3*; p. p. dppA3 | |  |
| **44** | **PAO1-phiKZ-48-I** | 21.9 |  |  |  |  |  | **●** |  | PhiKZ |  |  | *pilQ* |  | *yqjG* | *mexT* | p. p. dppA3; p. p. 16S rRNA | |  |
| **45** | **PAO1-phiKZ-72-C** | 12.6 |  |  |  |  |  | **●** |  | PhiKZ |  |  |  |  | *mexT* | | *wspF* | *dppA3*; p. p. dppA3; p. p. 16S rRNA |  |
| **46** | **PAO1-phiKZ-72-F** | 17.4 |  |  |  |  |  | **●** |  | PhiKZ |  |  |  |  | *vfr* | *mexT* | p. p. dppA3 | |  |
| **47** | **PAO1-phiKZ-P-C** | 25.1 |  |  |  |  |  | **●** |  |  |  |  |  | *fliN* | *mexT* | | p. p. dppA3; p. p. 16S rRNA | |  |
| **48** | **PAO1-phiKZ-P-F** | 30.2 |  |  |  |  |  | **●** |  |  |  |  |  |  | *mexT* | | p. p. dppA3; p. p. 16S rRNA | |  |
| **49** | **PAO1-KT28-phiKZ-72-E** | 42.2 |  |  | **●** |  |  | **●** |  | PhiKZ | *wzy* |  |  |  | *mexT* | | *dppA3*; p. p. dppA3; p. p. 16S rRNA | |  |
| **50** | **PAO1-KT28-phiKZ-72-G** | 46.6 |  |  | **●** |  |  | **●** |  | PhiKZ | *wzy* |  |  |  | *mexT* | | p. p. dppA3; p. p. 16S rRNA | |  |
| **51** | **PAO1-KT28-phiKZ-P-A** | 4.9 |  |  | **●** |  |  | **●** |  | PhiKZ | *wzy* |  |  |  | *mexT* | | p. p. dppA3; p. p. 16S rRNA | |  |
| **52** | **PAO1-KT28-phiKZ-P-C** | 9.7 |  |  | **●** |  |  | **●** |  | PhiKZ | *wzy* | *mucA* |  |  | *mexT* | | p. p. dppA3 | |  |
| **53** | **PAO1-KTN6-LUZ7-48-H** | 36.0 | **●** | **●** |  |  |  |  |  |  | *wzy* |  |  |  | *yqjG* | *mexT* | p. p. dppA3; p. p. 16S rRNA | |  |
| **54** | **PAO1-KTN6-LUZ7-P-G** | 21.9 | **●** | **●** |  |  |  |  |  |  |  |  |  |  | *mexT* | | p. p. dppA3; p. p. 16S rRNA | | 23 983 |
| **55** | **PAO1-KTN6-LUZ7-P-H** | 38.1 | **●** | **●** |  |  |  |  |  |  | *migA; wzy* |  |  |  | *mexT* | | PA5402 | p. p. dppA3. p. p. 16S rRNA |  |
| **56** | **PAO1-KTN6-LUZ7-P-N** | 17.4 | **●** | **●** |  |  |  |  |  |  |  |  |  |  | *mexT* | | p. p. dppA3 | | 412 171 |
| **57** | **PAO1-LUZ7-KTN4-48-J** | 4.3 | **●** |  |  |  | **●** |  |  | KTN4 |  |  |  |  | *mexT* | | p. p. dppA3 | |  |
| **58** | **PAO1-LUZ7-KTN4-72-A** | 8.8 | **●** |  |  |  | **●** |  |  | KTN4 |  |  | *pilZ* |  | *mexT* | | p. p. dppA3 | |  |
| **59** | **PAO1-LUZ7-KTN4-72-K** | 15.4 | **●** |  |  |  | **●** |  |  | KTN4 |  |  | *pilB* |  | *mexT* | | *lolE* | *dppA3*; p. p. dppA3; p. p. 16S rRNA |  |
| **60** | **PAO1-LUZ7-KTN4-72-M** | 9.4 | **●** |  |  |  | **●** |  |  | KTN4 |  |  |  |  | *mexT* | | *lolE* | *dppA3*; p. p. dppA3; p. p. 16S rRNA |  |
| **61** | **PAO1-LUZ7-KTN4-P-D** | 6.2 | **●** |  |  |  | **●** |  |  | KTN4 |  |  |  |  | *mexT* | | p. p. dppA3; p. p. 16S rRNA | |  |
| **62** | **PAO1-KTN6-KTN4-24-A** | 46.7 |  | **●** |  |  | **●** |  |  |  | *wzy* |  |  |  | *mexT* | | p. p. dppA3; p. p. 16S rRNA | |  |
| **63** | **PAO1-KTN6-KTN4-24-C** | 7.3 |  | **●** |  |  | **●** |  |  | KTN4 | *wzy* |  |  |  | *mexT* | | p. p. dppA3 ; p. p. 16S rRNA | |  |
| **64** | **PAO1-KTN6-KTN4-24-E** | 9.3 |  | **●** |  |  | **●** |  |  | KTN4 |  |  |  |  | *mexT* | | *dppA3* ; p. p. dppA3 | | 262 010 |
| **65** | **PAO1-KTN6-KTN4-24-H** | 15.1 |  | **●** |  |  | **●** |  |  |  |  |  |  |  | *mexT* | | p. p. dppA3 ; p. p. 16S rRNA | | 234 356 |
| **66** | **PAO1-KTN6-KTN4-48-C** | 5.8 |  | **●** |  |  | **●** |  |  | KTN4 |  |  |  |  | *mexT* | | p. p. dppA3 | | 314 003 |
| **67** | **PAO1-KTN6-KTN4-72-B** | 5.0 |  | **●** |  |  | **●** |  |  | KTN4 | *ssg* |  |  |  | *yqjG* | *mexT* | p. p. dppA3 | |  |
| **68** | **PAO1-KTN6-KTN4-P-B** | 46.7 |  | **●** |  |  | **●** |  |  |  | *wzy* |  |  |  | *mexT* | | p. p. dppA3 ; p. p. 16S rRNA | |  |
| **69** | **PAO1-KTN6-KTN4-P-H** | 40.9 |  | **●** |  |  | **●** |  |  |  | *ssg* |  |  |  | *mexT* | | p. p. dppA3 ; p. p. 16S rRNA | |  |
| **70** | **PAO1-KTN6-KTN4-P-N** | 4.3 |  | **●** |  |  | **●** |  |  |  |  |  | *fimU* |  | *mexT* | | p. p. dppA3 ; p. p. 16S rRNA | | 233 529 |
| **71** | **PAO1-KTN6-phiKZ-24-H** | 10.6 |  | **●** |  |  |  | **●** |  | PhiKZ |  |  |  |  | *yqjG; bifA* | *mexT* | p. p. dppA3. p. p. 16S rRNA | |  |
| **72** | **PAO1-KTN6-phiKZ-48-B** | 27.6 |  | **●** |  |  |  | **●** |  | PhiKZ | *wapH* |  | *pilR* |  | *mexT* | | p. p. dppA3 ; p. p. 16S rRNA | |  |
| **73** | **PAO1-KTN6-phiKZ-48-G** | 11.4 |  | **●** |  |  |  | **●** |  | PhiKZ |  |  |  |  | *yqjG* | *mexT* | p. p. dppA3; p. p. 16S rRNA | | 410 964 |
| **74** | **PAO1-KTN6-phiKZ-48-J** | 3.5 |  | **●** |  |  |  | **●** |  | PhiKZ |  |  |  |  | *mexT* | | *dppA3* ; p. p. 16S rRNA | | 46 891 |
| **75** | **PAO1-KTN6-phiKZ-48-L** | 12.6 |  | **●** |  |  |  | **●** |  | PhiKZ |  |  |  |  | *mexT* | | *dppA3* ; p. p. dppA3 ; p. p. 16S rRNA | |  |
| **76** | **PAO1-KTN6-phiKZ-72-C** | 7.0 |  | **●** |  |  |  | **●** |  | PhiKZ | *wzy* |  |  |  | *mexT* | | p. p. dppA3 | |  |
| **77** | **PAO1-KTN6-phiKZ-P-B** | 13.0 |  | **●** |  |  |  | **●** |  | PhiKZ |  |  |  |  | *mexT* | | p. p. dppA3 | |  |
| **78** | **PAO1-KTN6-phiKZ-P-K** | 5.0 |  | **●** |  |  |  | **●** |  |  |  |  |  |  | *yqjG* | *mexT* | p. p. dppA3 | | 333 643 |
| **79** | **PAO1-KTN6-phiKZ-P-U** | 17.4 |  | **●** |  |  |  | **●** |  |  |  |  |  |  | *mexT* | | p. p. dppA3 ; p. p. 16S rRNA | | 336 992 |
| **80** | **PAO1-KTN6-LUZ7-KTN4-24-A** | 9.3 | **●** | **●** |  |  | **●** |  |  | KTN4 |  |  |  |  | *mexT* | | p. p. dppA3 | |  |
| **81** | **PAO1-KTN6-LUZ7-KTN4-24-D** | 4.3 | **●** | **●** |  |  | **●** |  |  | KTN4 | *ssg* |  |  |  | *yqjG* | *mexT* | p. p. dppA3 | |  |
| **82** | **PAO1-KTN6-LUZ7-KTN4-24-F** | 40.7 | **●** | **●** |  |  | **●** |  |  | KTN4 | *ssg* |  |  |  | *yqjG* | *mexT* | p. p. dppA3; p. p. 16S rRNA | |  |
| **83** | **PAO1-KTN6-LUZ7-KTN4-24-H** | 14.3 | **●** | **●** |  |  | **●** |  |  |  |  |  |  |  | *yqjG* | *mexT* | p. p. dppA3 | | 359 043 |
| **84** | **PAO1-KTN6-LUZ7-KTN4-24-I** | 4.0 | **●** | **●** |  |  | **●** |  |  | KTN4 |  |  |  |  | *mexT* | | p. p. dppA3 ; p. p. 16S rRNA | |  |
| **85** | **PAO1-KTN6-LUZ7-KTN4-48-C** | 11.0 | **●** | **●** |  |  | **●** |  |  |  |  |  |  |  | *yqjG* | *mexT* | p. p. dppA3; p. p. 16S rRNA | | 544 729 |
| **86** | **PAO1-KTN6-LUZ7-KTN4-48-G** | 12.1 | **●** | **●** |  |  | **●** |  |  | KTN4 |  |  |  |  | *yqjG* | *mexT* | p. p. dppA3; p. p. 16S rRNA | |  |
| **87** | **PAO1-KTN6-LUZ7-KTN4-48-GI** | 3.6 | **●** | **●** |  |  | **●** |  |  | KTN4; LUZ7 |  |  |  |  | *mexT* | | p. p. dppA3 | | 467 093 |
| **88** | **PAO1-KTN6-LUZ7-KTN4-48-I** | 4.5 | **●** | **●** |  |  | **●** |  |  | KTN4 | *wbpK; ssg* |  | *pilB* |  | *yqjG* | *mexT* | p. p. dppA3 | |  |
| **89** | **PAO1-KTN6-LUZ7-KTN4-48-K** | 6.0 | **●** | **●** |  |  | **●** |  |  | KTN4 | *wzy* | *mucA* |  |  | *mexT* | | PA0429 | p. p. dppA3; p. p. 16S rRNA |  |
| **90** | **PAO1-KTN6-LUZ7-KTN4-72-L** | 4.5 | **●** | **●** |  |  | **●** |  |  | KTN4 |  |  |  |  | *mexT* | | p. p. dppA3 | | 420 856 |
| **91** | **PAO1-KTN6-LUZ7-KTN4-P-B** | 13.7 | **●** | **●** |  |  | **●** |  |  | KTN4 |  |  |  |  | *mexT* | | PA2911 | p. p. dppA3 |  |
| **92** | **PAO1-KTN6-LUZ7-KTN4-P-M** | 3.3 | **●** | **●** |  |  | **●** |  |  | KTN4 |  |  |  |  | *mexT* | | p. p. dppA3 | | 234 815 |
| **93** | **PAO1-KTN6-LUZ7-KTN4-P-W** | 16.2 | **●** | **●** |  |  | **●** |  |  |  |  | *mucA* |  |  | *mexT* | | p. p. dppA3 ; p. p. 16S rRNA | | 321 002 |
| **94** | **PAO1-KTN6-LUZ7-phiKZ-24-B** | 4.3 | **●** | **●** |  |  |  | **●** |  | PhiKZ |  |  |  |  | *mexT* | | p. p. dppA3 ; p. p. 16S rRNA | | 157 562 |
| **95** | **PAO1-KTN6-LUZ7-phiKZ-24-G** | 6.9 | **●** | **●** |  |  |  | **●** |  | PhiKZ |  |  |  |  | *mexT* | | *mviN* | p. p. dppA3; p. p. 16S rRNA |  |
| **96** | **PAO1-KTN6-LUZ7-phiKZ-24-H** | 7.9 | **●** | **●** |  |  |  | **●** |  | PhiKZ |  |  |  |  | *mexT* | | p. p. dppA3 ; p. p. 16S rRNA | | 310 097 |
| **97** | **PAO1-KTN6-LUZ7-phiKZ-24-L** | 2.8 | **●** | **●** |  |  |  | **●** |  | PhiKZ; KTN6 |  |  |  |  | *mexT* | | p.p. nagZ | *dppA3* ; p. p. dppA3 ; p. p. 16S rRNA |  |
| **98** | **PAO1-KTN6-LUZ7-phiKZ-48-D** | 35.6 | **●** | **●** |  |  |  | **●** |  |  | *wzy* |  |  |  | *mexT* | | p. p. dppA3 ; p. p. 16S rRNA | |  |
| **99** | **PAO1-KTN6-LUZ7-phiKZ-48-F** | 2.6 | **●** | **●** |  |  |  | **●** |  | PhiKZ; LUZ7 |  |  |  |  | *mexT* | | *dppA3* ; p. p. dppA3 | | 417 514 |
| **100** | **PAO1-KTN6-LUZ7-phiKZ-P-L** | 7.6 | **●** | **●** |  |  |  | **●** |  | PhiKZ | *wzy* |  |  |  | *mexT* | | PA2911 | p. p. dppA3; p. p. 16S rRNA |  |
| **101** | **PAO1-KTN6-LUZ7-phiKZ-P-O** | 28.2 | **●** | **●** |  |  |  | **●** |  |  |  | *algB* |  |  | *mexT* | | p. p. dppA3 ; p. p. 16S rRNA | |  |
| **102** | **PAO1-KTN6-LUZ7-phiKZ-P-R** | 4.5 | **●** | **●** |  |  |  | **●** |  |  |  |  |  |  | *mexT* | | p. p. dppA3 ; p. p. 16S rRNA | | 333 735 |
|  | **Total number**  **(frequency)** |  |  |  |  |  |  |  |  | **54 (56.8%)** | **33 (34.7%)** | **5 (5.2%)** | **15 (15.8%)** | **4 (4.2%)** | **24 (25.3%)** | | **11 (11.6%)** | | **22 (23.1%)** |

**Growth rate** estimated by measuring of optical density (OD_600_) kinetics expressed as the cumulated OD values. **Phage typing**: phage typing performed in the spot test could give provided three different results: resistance (navy blue); variable result (grey) and sensitivity (white). A dot in the middle of the cell indicates which phage was used for infection. **Phage DNA** found in the bacteria genome assembly analysis: green cells indicate the maintenance of particular phage DNA within the bacterial clone. **Mutations** detected using the software pipeline Snippy: yellow cells indicate mutations occurring exclusively under the phage pressure, while pink cells indicate spontaneous mutations (occurring also in control strains). **Large deletions** located by genome mapping: a custom python script and the software suite MEME v5.2.0.; light-red cells indicates deletions occurring in *galU* gene region.
