# Supplementary material for: Genome-driven elucidation of phage-host interplay and impact of phage resistance evolution on bacterial fitness
Source: ISME J. 2021 Aug 31;16(2):533–42. doi: 10.1038/s41396-021-01096-5 (PMC8776877; doi:10.1038/s41396-021-01096-5)
Supplement: Supplementary file 3 — Table S3 [file 41396_2021_1096_MOESM3_ESM.docx]

**Table S3.** Characteristics of clinical *P. aeruginosa* controls and phage-resistant isolates

| **#** | **ISOLATE** |  | **PHAGE SUSCEPTIBILITY** | | | | | | | **PHAGE DNA** | **REGION AFFECTED WITH MUTATION** | | | | | | | | **LARGE DELETION [bp]** |
| --- | --- | --- | --- | --- | --- | --- | --- | --- | --- | --- | --- | --- | --- | --- | --- | --- | --- | --- | --- |
|  |  |  | **LPS phages** | | | **T4P phages** | | |  |  |  |  |  |  |  |  |  |  |  |
|  |  | **CUMULATED OD_600_ VALUES**  **OF 18H** | **LUZ7** | **KTN6** | **KT28** | **LUZ19** | **KTN4** | **phiKZ** | **PA5oct** |  | **LPS** | **EPS** | **T4P** | **Flagella** | **Global** | | **Others** | |  |
| **1** | **A5803** | 44.6 |  |  |  |  |  |  |  |  |  |  |  |  |  | |  | |  |
| **2** | **A5803-K-24-A** | 49.0 |  |  |  |  |  |  |  |  |  |  |  |  |  | |  | |  |
| **3** | **A5803-K-24-B** | 46.1 |  |  |  |  |  |  |  |  |  |  |  |  |  | |  | |  |
| **4** | **A5803-K-24-C** | 46.9 |  |  |  |  |  |  |  |  |  |  |  |  |  | |  | |  |
| **5** | **A5803-LUZ7-24-B** | 46.9 | **●** |  |  |  |  |  |  |  |  |  |  |  | *cpdA* | | PA2911 | |  |
| **6** | **A5803-LUZ7-48-A** | 21.0 | **●** |  |  |  |  |  |  |  |  |  |  |  |  | | PA3256 | | 199 182 |
| **7** | **A5803-LUZ7-48-C** | 49.5 | **●** |  |  |  |  |  |  |  |  |  |  |  | *cpdA* | | PA2911 | |  |
| **8** | **A5803-LUZ7-72-A** | 46.3 | **●** |  |  |  |  |  |  |  |  |  |  |  | *tRNA-Thr* | | PA2911 | |  |
| **9** | **A5803-LUZ7-72-B** | 47.8 | **●** |  |  |  |  |  |  |  |  |  |  |  |  | | PA2911 | |  |
| **10** | **A5803-KTN6-72-A** | 46.0 |  | **●** |  |  |  |  |  |  |  |  |  |  | *cpdA; mvfR* | |  | |  |
| **11** | **A5803-KTN6-72-E** | 47.9 |  | **●** |  |  |  |  |  |  |  |  |  |  |  | |  | |  |
| **12** | **A5803-KTN6-72-F** | 45.9 |  | **●** |  |  |  |  |  |  |  |  |  |  |  | |  | |  |
| **13** | **A5803-KTN6-72-H** | 47.3 |  | **●** |  |  |  |  |  |  |  |  |  |  | *cpdA; mvfR* | | PA1791 | |  |
| **14** | **A5803-KTN6-72-K** | 46.2 |  | **●** |  |  |  |  |  |  |  |  |  |  | *cpdA; spuF* | |  | |  |
| **15** | **A5803-phiKZ-24-C** | 47.3 |  |  |  |  |  | **●** |  |  |  |  |  |  | *cpdA* | |  | |  |
| **16** | **A5803-phiKZ-48-A** | 44.9 |  |  |  |  |  | **●** |  |  |  |  |  |  | *cpdA* | | p. p. PA2422 | |  |
| **17** | **A5803-phiKZ-48-B** | 45.5 |  |  |  |  |  | **●** |  |  |  |  |  |  | *cpdA; spuF* | |  | |  |
| **18** | **A5803-phiKZ-72-A** | 45.8 |  |  |  |  |  | **●** |  |  |  |  | *pilQ* |  |  | |  | |  |
| **19** | **A5803-phiKZ-72-D** | 44.3 |  |  |  |  |  | **●** |  |  |  |  |  |  | *cpdA* | |  | |  |
| **20** | **A5803-PA5oct-24-A** | 46.8 |  |  |  |  |  |  | **●** |  |  |  |  |  |  | |  | |  |
| **21** | **A5803-PA5oct-24-B** | 46.2 |  |  |  |  |  |  | **●** |  |  |  |  |  | *cpdA* | |  | |  |
| **22** | **A5803-PA5oct-24-C** | 47.3 |  |  |  |  |  |  | **●** |  |  |  |  |  |  | |  | |  |
| **23** | **A5803-PA5oct-48-A** | 45.6 |  |  |  |  |  |  | **●** |  |  |  |  |  |  | |  | |  |
| **24** | **A5803-PA5oct-72-C** | 46.5 |  |  |  |  |  |  | **●** |  |  |  |  |  | *cpdA* | |  | |  |
| **25** | **A5803-KTN6-LUZ7-24-K** | 32.9 | **●** | **●** |  |  |  |  |  |  |  |  |  |  |  | | PA2911 | | 244 307 |
| **26** | **A5803-KTN6-LUZ7-24-M** | 41.2 | **●** | **●** |  |  |  |  |  |  |  |  |  |  | *tRNA-Thr* | |  | |  |
| **27** | **A5803-KTN6-LUZ7-48-E** | 17.3 | **●** | **●** |  |  |  |  |  |  |  |  |  |  |  | | p.p. yneJ | | 254 739 |
| **28** | **A5803-KTN6-LUZ7-72-A** | 41.6 | **●** | **●** |  |  |  |  |  |  |  |  |  |  | *cpdA* | | PA2911; *pchR* | |  |
| **29** | **A5803-KTN6-LUZ7-72-L** | 44.2 | **●** | **●** |  |  |  |  |  |  |  |  |  |  | *cpdA* | |  | |  |
| **30** | **A5803-KTN6-phiKZ-24-H** | 41.3 |  | **●** |  |  |  | **●** |  |  |  | *mucA* |  |  | *cpdA* | |  | |  |
| **31** | **A5803-KTN6-phiKZ-24-L** | 42.3 |  | **●** |  |  |  | **●** |  |  |  |  |  |  |  | |  | |  |
| **32** | **A5803-KTN6-phiKZ-48-D** | 33.1 |  | **●** |  |  |  | **●** |  |  |  |  | *pilF* |  | *cpdA* | |  | |  |
| **33** | **A5803-KTN6-phiKZ-48-K** | 42.2 |  | **●** |  |  |  | **●** |  |  |  |  |  |  |  | |  | |  |
| **34** | **A5803-KTN6-phiKZ-48-O** | 41.8 |  | **●** |  |  |  | **●** |  |  |  |  |  |  | *cpdA* | |  | |  |
| **35** | **A5803-KTN6-LUZ7-phiKZ-24-T** | 18.7 | **●** | **●** |  |  |  | **●** |  |  |  |  |  |  |  | |  | | 383 693 |
| **36** | **A5803-KTN6-LUZ7-phiKZ-24-F** | 44.9 | **●** | **●** |  |  |  | **●** |  |  |  |  |  |  | *cpdA; spuF* | | PA2911 | |  |
| **37** | **A5803-KTN6-LUZ7-phiKZ-24-I** | 13.2 | **●** | **●** |  |  |  | **●** |  |  |  |  | *pilN* |  | *tRNA-Thr* | |  | | 245 669 |
| **38** | **A5803-KTN6-LUZ7-phiKZ-72-L** | 25.3 | **●** | **●** |  |  |  | **●** |  |  | *galU* |  |  |  |  | |  | |  |
| **39** | **A5803-KTN6-LUZ7-phiKZ-72-R** | 45.8 | **●** | **●** |  |  |  | **●** |  |  |  |  |  |  |  | |  | |  |
|  | **Total number (frequency)** |  |  |  |  |  |  |  |  | **0 (0%)** | **1 (2.9%)** | **1 (2.9%)** | **3 (8.6%)** | **0 (0%)** | **25 (71.4%)** | | **12 (34.3%)** | | **5 (14.3%)** |
| **40** | **AA43** | 23.0 |  |  |  |  |  |  |  |  |  |  |  |  |  | |  | |  |
| **41** | **AA43-K-72-A** | 21.8 |  |  |  |  |  |  |  |  |  |  |  |  |  | |  | |  |
| **42** | **AA43-K-72-B** | 21.7 |  |  |  |  |  |  |  |  |  |  |  |  |  | |  | |  |
| **43** | **AA43-K-72-C** | 17.9 |  |  |  |  |  |  |  |  |  |  |  |  |  | |  | |  |
| **44** | **AA43-LUZ7-24-B** | 21.1 | **●** |  |  |  |  |  |  |  |  |  |  |  |  | |  | |  |
| **45** | **AA43-LUZ7-48-A** | 23.9 | **●** |  |  |  |  |  |  |  |  |  |  |  |  | | PA2911 | |  |
| **46** | **AA43-LUZ7-48-B** | 21.3 | **●** |  |  |  |  |  |  |  | *wzy* |  |  |  |  | | PA2911 | |  |
| **47** | **AA43-LUZ7-72-A** | 6.5 | **●** |  |  |  |  |  |  | LUZ7 |  |  |  |  |  | | *gltS; pscP* | |  |
| **48** | **AA43-LUZ7-72-D** | 20.1 | **●** |  |  |  |  |  |  |  |  |  |  |  |  | |  | |  |
| **49** | **AA43-KTN6-48-G** | 19.4 |  | **●** |  |  |  |  |  | KTN6 |  |  |  |  | *fha1* | |  | |  |
| **50** | **AA43-KTN6-48-K** | 14.8 |  | **●** |  |  |  |  |  | KTN6 |  |  |  |  |  | |  | |  |
| **51** | **AA43-KTN6-72-A** | 20.7 |  | **●** |  |  |  |  |  |  |  |  |  |  |  | |  | |  |
| **52** | **AA43-KTN6-72-G** | 21.4 |  | **●** |  |  |  |  |  |  |  |  |  |  |  | |  | |  |
| **53** | **AA43-KTN6-72-I** | 12.3 |  | **●** |  |  |  |  |  | KTN6 | *migA; wzy* |  |  |  | *ftsH* | |  | |  |
| **54** | **AA43-phiKZ-24-C** | 16.0 |  |  |  |  |  | **●** |  |  |  |  |  |  |  | |  | |  |
| **55** | **AA43-phiKZ-48-A** | 15.8 |  |  |  |  |  | **●** |  |  |  |  |  |  |  | |  | |  |
| **56** | **AA43-phiKZ-48-B** | 16.5 |  |  |  |  |  | **●** |  |  |  |  | *pilR* |  |  | |  | |  |
| **57** | **AA43-phiKZ-72-A** | 19.3 |  |  |  |  |  | **●** |  |  |  |  | *pilR* |  |  | |  | |  |
| **58** | **AA43-phiKZ-72-C** | 22.1 |  |  |  |  |  | **●** |  |  |  |  |  |  |  | |  | |  |
| **59** | **AA43-PA5oct-24-A** | 15.5  /99999/* |  |  |  |  |  |  | **●** |  |  |  |  |  |  | |  | | 227 848 |
| **60** | **AA43-PA5oct-24-C** | 3.4 |  |  |  |  |  |  | **●** | PA5oct |  |  |  |  |  | |  | |  |
| **61** | **AA43-PA5oct-48-C** | 22.9 |  |  |  |  |  |  | **●** |  | *wapH* |  |  |  |  | |  | |  |
| **62** | **AA43-PA5oct-48-D** | 3.6 |  |  |  |  |  |  | **●** | PA5oct |  |  |  |  |  | |  | |  |
| **63** | **AA43-PA5oct-72-A** | 21.3 |  |  |  |  |  |  | **●** |  |  |  |  |  |  | |  | |  |
| **64** | **AA43-KTN6-LUZ7-24-A** | 19.4 | **●** | **●** |  |  |  |  |  |  |  |  |  |  |  | |  | |  |
| **65** | **AA43-KTN6-LUZ7-48-C** | 18.3 | **●** | **●** |  |  |  |  |  |  | *wzy* |  |  |  |  | | PA2911 | |  |
| **66** | **AA43-KTN6-LUZ7-48-O** | 21.3 | **●** | **●** |  |  |  |  |  |  |  |  |  |  |  | |  | |  |
| **67** | **AA43-KTN6-LUZ7-72-A** | 23.1 | **●** | **●** |  |  |  |  |  |  | *wzy* |  |  |  |  | | PA2911 | |  |
| **68** | **AA43-KTN6-LUZ7-72-K** | 14.9 | **●** | **●** |  |  |  |  |  |  |  |  |  |  |  | | *wspF* | |  |
| **69** | **AA43-KTN6-phiKZ-24-A** | 3.3 |  | **●** |  |  |  | **●** |  | KTN6 |  |  | *pilT* |  |  | |  | |  |
| **70** | **AA43-KTN6-phiKZ-24-G** | 12.5 |  | **●** |  |  |  | **●** |  | KTN6 |  |  | *pilR* |  |  | |  | |  |
| **71** | **AA43-KTN6-phiKZ-24-L** | 14.1 |  | **●** |  |  |  | **●** |  |  |  |  | *h. pilB* |  |  | |  | |  |
| **72** | **AA43-KTN6-phiKZ-72--E** | 14.8 |  | **●** |  |  |  | **●** |  |  |  |  |  |  |  | | PA2438 | |  |
| **73** | **AA43-KTN6-phiKZ-72-N** | 20.3 |  | **●** |  |  |  | **●** |  |  |  |  |  |  |  | | *wspF* | |  |
| **74** | **AA43-KTN6-LUZ7-phiKZ-24-B** | 20.9 | **●** | **●** |  |  |  | **●** |  |  |  |  |  |  |  | |  | |  |
| **75** | **AA43-KTN6-LUZ7-phiKZ-72-B** | 13.5 | **●** | **●** |  |  |  | **●** |  | LUZ7 |  |  |  |  |  | | PA5438 | | 92 207 |
| **76** | **AA43-KTN6-LUZ7-phiKZ-72-C** | 8.1 | **●** | **●** |  |  |  | **●** |  |  |  |  |  |  |  | | *wspF* | | 378.656 |
| **77** | **AA43-KTN6-LUZ7-phiKZ-72-L** | 2.6 | **●** | **●** |  |  |  | **●** |  | phiKZ |  |  |  |  | *yfiN* | |  | |  |
| **78** | **AA43-KTN6-LUZ7-phiKZ-72-S** | 16.0 | **●** | **●** |  |  |  | **●** |  | phiKZ |  |  |  |  |  | |  | |  |
|  | **Total number (frequency)** |  |  |  |  |  |  |  |  | **11 (31.4%)** | **6 (17.1%)** | **0 (0%)** | **5 (14.3%)** | **0 (0%)** | **3 (8.6%)** | | **11 (31.4%)** | | **3 (8.6%)** |
| **79** | **CHA** | 37.9 |  |  |  |  |  |  |  |  |  |  |  |  |  | |  | |  |
| **80** | **CHA-K-72-A** | 41.7 |  |  |  |  |  |  |  |  |  |  |  |  |  | | *wspF* | |  |
| **81** | **CHA-K-72-B** | 38.0 |  |  |  |  |  |  |  |  |  |  |  |  |  | | *wspA* | |  |
| **82** | **CHA-K-72-C** | 42.9 |  |  |  |  |  |  |  |  |  |  |  |  |  | | *wspF* | |  |
| **83** | **CHA-LUZ7-24-A** | 29.8 | **●** |  |  |  |  |  |  |  |  |  |  |  | *morA* | |  | |  |
| **84** | **CHA-LUZ7-24-B** | 44.6 | **●** |  |  |  |  |  |  |  |  |  | *pilR* |  |  | | h. gene | *wspF* |  |
| **85** | **CHA-LUZ7-48-D** | 39.0 | **●** |  |  |  |  |  |  |  | *wbpA* |  |  |  |  | | PA4772 | *wspA* |  |
| **86** | **CHA-LUZ7-72-A** | 36.1 | **●** |  |  |  |  |  |  |  |  |  |  |  | *morA* | | h. gene | |  |
| **87** | **CHA-LUZ7-72-B** | 5.0 | **●** |  |  |  |  |  |  |  |  |  |  |  |  | | *wspA* | |  |
| **88** | **CHA-KTN6-48-D** | 30.6 |  | **●** |  |  |  |  |  |  |  |  |  |  |  | | *wspA* | |  |
| **89** | **CHA-KTN6-72-C** | 35.7 |  | **●** |  |  |  |  |  |  |  |  |  |  | *morA* | |  | |  |
| **90** | **CHA-KTN6-72-D** | 36.5 |  | **●** |  |  |  |  |  |  |  |  |  |  | *morA* | | p. p. mvaU | |  |
| **91** | **CHA-KTN6-72-E** | 46.1 |  | **●** |  |  |  |  |  |  |  |  | *h. pilC* |  | *parS; dipA* | |  | |  |
| **92** | **CHA-KTN6-72-R** | 45.3 |  | **●** |  |  |  |  |  |  |  |  |  |  |  | | h. gene | *wspF* |  |
| **93** | **CHA-phiKZ-24-A** | 30.8 |  |  |  |  |  | **●** |  |  |  |  |  |  |  | |  | |  |
| **94** | **CHA-phiKZ-24-B** | 44.6 |  |  |  |  |  | **●** |  |  |  |  |  |  |  | | *wspF* | |  |
| **95** | **CHA-phiKZ-48-B** | 44.1 |  |  |  |  |  | **●** |  |  |  |  | *pilB* |  | *dipA* | |  | |  |
| **96** | **CHA-phiKZ-72-A** | 46.3 |  |  |  |  |  | **●** |  |  |  |  | *pilO* |  | *morA; gidB* | |  | |  |
| **97** | **CHA-phiKZ-72-B** | 9.8 |  |  |  |  |  | **●** |  |  |  |  |  |  |  | | *wspF* | |  |
| **98** | **CHA-PA5oct-24-A** | 46.5 |  |  |  |  |  |  | **●** |  |  |  | *pilQ* |  | *dipA* | |  | |  |
| **99** | **CHA-PAoct-24-B** | 29.2 |  |  |  |  |  |  | **●** |  |  |  |  |  |  | | *wspF* | |  |
| **100** | **CHA-PA5oct-48-C** | 39.9 |  |  |  |  |  |  | **●** |  |  |  |  |  |  | | *wspF* | |  |
| **101** | **CHA-PA5oct-72-A** | 22.9 |  |  |  |  |  |  | **●** |  |  |  |  |  |  | | *wspA* | |  |
| **102** | **CHA-PA5oct-72-C** | 46.3 |  |  |  |  |  |  | **●** |  |  |  | *pilR* |  |  | | *wspF* | |  |
| **103** | **CHA-KTN6-LUZ7-24-E** | 20.9 | **●** | **●** |  |  |  |  |  |  |  |  |  |  |  | |  | |  |
| **104** | **CHA-KTN6-LUZ7-24-F** | 31.9 | **●** | **●** |  |  |  |  |  |  | *wapH* |  |  |  | *morA* | |  | |  |
| **105** | **CHA-KTN6-LUZ7-24-P** | 20.8 | **●** | **●** |  |  |  |  |  |  |  | *algU* |  |  |  | |  | |  |
| **106** | **CHA-KTN6-LUZ7-48-M** | 31.1 | **●** | **●** |  |  |  |  |  |  |  |  |  |  |  | | h. gene | *wspA* |  |
| **107** | **CHA-KTN6-LUZ7-48-N** | 42.5 | **●** | **●** |  |  |  |  |  |  |  |  | *pilQ* |  |  | | *wspA* | | 15 126 |
| **108** | **CHA-KTN6-phiKZ-48-F** | 43.1 |  | **●** |  |  |  | **●** |  |  |  |  | *pilM* |  | *parS* | | *wspF* | |  |
| **109** | **CHA-KTN6-phiKZ-48-J** | 18.3 |  | **●** |  |  |  | **●** |  | KTN6 |  |  |  |  |  | | *wspF* | |  |
| **110** | **CHA-KTN6-phiKZ-48-P** | 23.0 |  | **●** |  |  |  | **●** |  |  |  |  | *pilT* |  |  | |  | |  |
| **111** | **CHA-KTN6-phiKZ-48-R** | 29.6 |  | **●** |  |  |  | **●** |  |  |  |  | *pilT* |  |  | |  | |  |
| **112** | **CHA-KTN6-phiKZ-72-K** | 23.2 |  | **●** |  |  |  | **●** |  |  |  |  |  |  |  | | *wspA* | |  |
| **113** | **CHA-KTN6-LUZ7-phiKZ-24-C** | 42.9 | **●** | **●** |  |  |  | **●** |  |  |  |  | *pilT* |  |  | |  | |  |
| **114** | **CHA-KTN6-LUZ7-phiKZ-24-L** | 30.6 | **●** | **●** |  |  |  | **●** |  |  |  | *algU* |  |  |  | |  | |  |
| **115** | **CHA-KTN6-LUZ7-phiKZ-48-D** | 31.5 | **●** | **●** |  |  |  | **●** |  |  |  |  |  |  |  | |  | |  |
| **116** | **CHA-KTN6-LUZ7-phiKZ-72-B** | 24.6 | **●** | **●** |  |  |  | **●** |  |  |  |  | *pilT* |  |  | |  | |  |
| **117** | **CHA-KTN6-LUZ7-phiKZ-72-R** | 28.0 | **●** | **●** |  |  |  | **●** |  |  |  |  |  |  |  | | *wspA* | |  |
|  | **Total number (frequency)** |  |  |  |  |  |  |  |  | **1 (2.9%)** | **2 (5.7%)** | **2 (5.7%)** | **12 (34.3%)** | **0 (0%)** | **12 (34.2%)** | | **6 (17.1%)** | | **0 (0%)** |
| **118** | **PAK** | 23.6 |  |  |  |  |  |  |  |  |  |  |  |  |  | |  | |  |
| **119** | **PAK-K-72-A** | 25.9 |  |  |  |  |  |  |  |  |  |  |  |  |  | |  | |  |
| **120** | **PAK-K-72-B** | 23.7 |  |  |  |  |  |  |  |  |  |  |  |  |  | |  | |  |
| **121** | **PAK-K-72-C** | 25.7 |  |  |  |  |  |  |  |  |  |  |  |  | *dipA* | |  | |  |
| **122** | **PAK-LUZ7-24-C** | 20.6 | **●** |  |  |  |  |  |  |  |  |  |  |  |  | | PA1943 | |  |
| **123** | **PAK-LUZ7-48-C** | 16.4 | **●** |  |  |  |  |  |  |  |  |  |  |  |  | |  | | 370 051 |
| **124** | **PAK-LUZ7-48-D** | 19.5 | **●** |  |  |  |  |  |  |  |  |  |  |  | *parS* | | PA2911 | |  |
| **125** | **PAK-LUZ7-72-A** | 20.7 | **●** |  |  |  |  |  |  |  |  |  |  |  |  | |  | |  |
| **126** | **PAK-LUZ7-72-B** | 20.0 | **●** |  |  |  |  |  |  |  |  |  |  |  |  | |  | |  |
| **127** | **PAK-KTN6-72-B** | 15.9 |  | **●** |  |  |  |  |  |  |  |  |  | *flhA* |  | |  | |  |
| **128** | **PAK-KTN6-72-C** | 27.6 |  | **●** |  |  |  |  |  |  |  |  | *pilT* |  |  | |  | |  |
| **129** | **PAK-KTN6-72-D** | 22.2 |  | **●** |  |  |  |  |  |  |  |  |  |  |  | | PA2911 | |  |
| **130** | **PAK-KTN6-72-F** | 23.9 |  | **●** |  |  |  |  |  |  |  |  |  |  | *parS* | |  | |  |
| **131** | **PAK-KTN6-72-K** | 24.2 |  | **●** |  |  |  |  |  |  |  |  |  |  | *parS* | *dipA* |  | |  |
| **132** | **PAK-phiKZ-24-A** | 5.3 |  |  |  |  |  | **●** |  | phiKZ |  |  | *pilB* |  |  | |  | |  |
| **133** | **PAK-phiKZ-48-A** | 20.4 |  |  |  |  |  | **●** |  |  |  |  |  |  |  | |  | |  |
| **134** | **PAK-phiKZ-48-B** | 6.4 |  |  |  |  |  | **●** |  | phiKZ |  |  | *pilJ* |  |  | |  | |  |
| **135** | **PAK-phiKZ-72-B** | 5.1 |  |  |  |  |  | **●** |  | phiKZ |  |  |  |  |  | |  | |  |
| **136** | **PAK-phiKZ-72-C** | 23.7 |  |  |  |  |  | **●** |  | phiKZ |  |  |  |  | *dipA* | |  | |  |
| **137** | **PAK-PA5oct-24-A** | 22.3 |  |  |  |  |  |  | **●** |  |  |  |  |  |  | |  | |  |
| **138** | **PAK-PA5oct-24-B** | 21.3 |  |  |  |  |  |  | **●** |  |  |  |  |  |  | |  | |  |
| **139** | **PAK-PA5oct-24-C** | 23.7 |  |  |  |  |  |  | **●** |  |  |  |  |  |  | |  | |  |
| **140** | **PAK-PA5oct-48-A** | 25.0 |  |  |  |  |  |  | **●** |  |  |  |  |  |  | |  | |  |
| **141** | **PAK-PA5oct-48-B** | 20.6 |  |  |  |  |  |  | **●** |  |  |  |  |  |  | |  | |  |
| **142** | **PAK-KTN6-LUZ7-24-C** | 35.5 | **●** | **●** |  |  |  |  |  |  |  |  |  |  |  | |  | |  |
| **143** | **PAK-KTN6-LUZ7-24-N** | 15.6 | **●** | **●** |  |  |  |  |  |  |  |  |  |  |  | |  | | 30 024 |
| **144** | **PAK-KTN6-LUZ7-48-C** | 31.5 | **●** | **●** |  |  |  |  |  |  |  |  | *pilT* |  |  | | PA2911 | |  |
| **145** | **PAK-KTN6-LUZ7-48-F** | 17.0 | **●** | **●** |  |  |  |  |  |  |  |  |  |  |  | |  | | 369 505 |
| **146** | **PAK-KTN6-LUZ7-48-R** | 14.5 | **●** | **●** |  |  |  |  |  |  |  |  |  |  |  | | PA2911 | | 248 827 |
| **147** | **PAK-KTN6-phiKZ-24-E** | 16.7 |  | **●** |  |  |  | **●** |  |  |  |  |  |  |  | |  | |  |
| **148** | **PAK-KTN6-phiKZ-48-C** | 16.3 |  | **●** |  |  |  | **●** |  |  |  |  | *pilS* |  | *dipA* | |  | |  |
| **149** | **PAK-KTN6-phiKZ-72-C** | 7.8 |  | **●** |  |  |  | **●** |  | phiKZ |  |  | *pilB* |  | *dipA* | |  | |  |
| **150** | **PAK-KTN6-phiKZ-72-F** | 4.5 |  | **●** |  |  |  | **●** |  | phiKZ. KTN6 |  |  | *pilT* |  |  | |  | |  |
| **151** | **PAK-KTN6-phiKZ-72-G** | 4.6 |  | **●** |  |  |  | **●** |  |  |  |  | *pilP* |  | *dipA* | |  | | 95 131 |
| **152** | **PAK-KTN6-LUZ7-phiKZ-48-H** | 18.1 | **●** | **●** |  |  |  | **●** |  |  |  |  |  |  |  | | *lolE* | |  |
| **153** | **PAK-KTN6-LUZ7-phiKZ-48-P** | 16.4 | **●** | **●** |  |  |  | **●** |  |  |  |  |  |  | *tpbB* | |  | |  |
| **154** | **PAK-KTN6-LUZ7-phiKZ-48-R** | 10.3 | **●** | **●** |  |  |  | **●** |  |  |  |  |  |  |  | | *wspA* | | 276 616 |
| **155** | **PAK-KTN6-LUZ7-phiKZ-72-E** | 18.4 | **●** | **●** |  |  |  | **●** |  |  |  |  | *pilT* |  |  | | PA2911 | |  |
| **156** | **PAK-KTN6-LUZ7-phiKZ-72-J** | 3.6 | **●** | **●** |  |  |  | **●** |  | phiKZ |  | *mucA* |  |  | *dipA* | |  | |  |
|  | **Total number (frequency)** |  |  |  |  |  |  |  |  | **8 (22.9%)** | **0 (0%)** | **1 (2.9%)** | **9 (25.7%)** | **1 (2.9%)** | **4 (11.4%)** | | **8 (22.9%)** | | **6 (17.1%)** |

**Growth rate** estimated by measuring of optical density (OD600) kinetics expressed as the cumulated OD values. **Phage typing**: phage typing performed in the spot test could give provided three different results: resistance (navy blue); variable result (grey) and sensitivity (white). A dot in the middle of the cell indicates which phage was used for infection. **Phage DNA** found in the bacteria genome assembly analysis: green cells indicate the maintenance of particular phage DNA within the bacterial clone. **Mutations** detected using the software pipeline Snippy. Yellow cells indicate mutations occurring exclusively under the phage pressure, while pink cells indicate spontaneous mutations (occurring also in control strains). **Large deletions** located by genome mapping: a custom python script and the software suite MEME v5.2.0.; light-red cells indicates deletions occurring in *galU* gene region.
