# Supplementary material for: Genome-driven elucidation of phage-host interplay and impact of phage resistance evolution on bacterial fitness
Source: ISME J. 2021 Aug 31;16(2):533–42. doi: 10.1038/s41396-021-01096-5 (PMC8776877; doi:10.1038/s41396-021-01096-5)
Supplement: Supplementary file 5 — Table S5 [file 41396_2021_1096_MOESM5_ESM.docx]

**Table S5.** Prophage elements within genomes of tested wild-type *P. aeruginosa* strains (according to PHASTER)

| **Strain** | **Prophage** | **DNA type** | **Taxonomy** | **Genome location** | **Length [bp]** | **Phage / total proteins** | **Completeness*** | **Most similar phage** | **Genbank** |
| --- | --- | --- | --- | --- | --- | --- | --- | --- | --- |
| **A5803** | **A5803#1** | **dsDNA** | ***Siphoviridae*** | **1166266-1207473** | **41,207** | **53/53** | **intact** | ***Pseudomonas* phage JBD25** | **NC_027992** |
|  | A5803#2 | dsDNA | *Siphoviridae* | 1432807-1474458 | 41,661 | 50/58 | questionable | *Pseudomonas* phage MD8 | NC_031091 |
|  | **A5803#3** | **dsDNA** | ***Myoviridae*** | **1538104-1564476** | **26,372** | **25/33** | **intact** | ***Escherichia* phage vB_EcoM_ep3** | **NC_025430** |
|  | **A5803#4** | **dsDNA** | ***Siphoviridae*** | **2546841-2604957** | **58,116** | **57/62** | **intact** | ***Pseudomonas* phage phi297** | **NC_016762** |
|  | **A5803#5** | **dsDNA** | ***Myoviridae*** | **5037092-5075814** | **38,722** | **46/48** | **intact** | ***Pseudomonas* phage phiCTX** | **NC_003278** |
|  | A5803#6 | ssDNA | *Inoviridae* | 5155631-5174796 | 19,165 | 11/12 | questionable | *Pseudomonas* phage Pf1 | NC_001331 |
|  | A5803#7 | ssDNA | *Siphoviridae* | 6035241-6073425 | 38,184 | 44/45 | questionable | *Pseudomonas* phage JBD67 | NC_042135 |
| **CHA** | **CHA#1** | **dsDNA** | ***Siphoviridae*** | **2447508-2487948** | **40,440** | **50/50** | **intact** | ***Pseudomonas* phage JBD18** | **NC_027986** |
|  | **CHA#2** | **dsDNA** | ***Myoviridae*** | **2604637-2643572** | **38,935** | **37/46** | **intact** | ***Pseudomonas* phage phiCTX** | **NC_003278** |
|  | CHA#3 | ssDNA | *Inoviridae* | 4338309-4356579 | 18,270 | 7/8 | incomplete | *Pseudomonas* phage Pf1 | NC_001331 |
|  | CHA#4 | ssDNA | *Inoviridae* | 5616455-5631581 | 15,126 ** | 8/8 | questionable* | *Pseudomonas* phage Pf1 | NC_001331 |
| **PAK** | **PAK#1** | **ssDNA** | ***Inoviridae*** | **5351074-5363016** | **11,942** | **10/10** | **intact** | ***Pseudomonas* phage Pf1** | **NC_001331** |
| **PAO1** | PAO#1 | ssDNA | *Inoviridae* | 780995-796776 | 15,781 | 8/8 | questionable | *Pseudomonas* phage Pf1 | NC_001331 |

* Completeness according to PHASTER algorithms

** Manual data curation
